# Fetal and obstetrics manifestations of mitochondrial diseases

**DOI:** 10.1186/s12967-024-05633-6

**Published:** 2024-09-23

**Authors:** Alessia Adelizzi, Anastasia Giri, Alessia Di Donfrancesco, Simona Boito, Alessandro Prigione, Emanuela Bottani, Valentina Bollati, Valeria Tiranti, Nicola Persico, Dario Brunetti

**Affiliations:** 1 Unit of Medical Genetics and Neurogenetics, Fondazione IRCCS Istituto Neurologico “Carlo Besta”, Milan, Italy; 2 Fetal Medicine and Surgery Service, Ospedale Maggiore Policlinico, Fondazione IRCCS Ca’ Granda, Milan, Italy; 3 Department of General Pediatrics, Neonatology and Pediatric Cardiology, Medical Faculty, University Hospital Düsseldorf, Heinrich Heine University Düsseldorf, Düsseldorf, Germany; 4 Department of Diagnostics and Public Health, University of Verona, Verona, 37124 Italy; 5 Dipartimento di Scienze Cliniche e di Comunità, Dipartimento di Eccellenza, University of Milan, Milan, 2023-2027 Italy

## Abstract

During embryonic and neonatal development, mitochondria have essential effects on metabolic and energetic regulation, shaping cell fate decisions and leading to significant short- and long-term effects on embryonic and offspring health. Therefore, perturbation on mitochondrial function can have a pathological effect on pregnancy. Several shreds of evidence collected in preclinical models revealed that severe mitochondrial dysfunction is incompatible with life or leads to critical developmental defects, highlighting the importance of correct mitochondrial function during embryo-fetal development. The mechanism impairing the correct development is unknown and may include a dysfunctional metabolic switch in differentiating cells due to decreased ATP production or altered apoptotic signalling. Given the central role of mitochondria in embryonic and fetal development, the mitochondrial dysfunction typical of Mitochondrial Diseases (MDs) should, in principle, be detectable during pregnancy. However, little is known about the clinical manifestations of MDs in embryonic and fetal development. In this manuscript, we review preclinical and clinical evidence suggesting that MDs may affect fetal development and highlight the fetal and maternal outcomes that may provide a wake-up call for targeted genetic diagnosis.

## Introduction

### Mitochondria: an overview

Mitochondria are cellular organelles commonly considered “the powerhouses” of the cell, as their primary function is to produce metabolic energy through oxidative phosphorylation (OXPHOS). Beyond this essential function, these organelles play a central role in other aspects of cell life and death, including heat production, apoptosis, metabolite-mediated modulation of gene expression, calcium and free radical signalling. Hence, mitochondria can act as gatekeepers of cellular homeostasis and regulate stem cell fate decisions [1], impacting embryogenesis and organogenesis [2–5].

Although mitochondrial contribution was well studied in physiological and pathological conditions after birth, little is known about mitochondrial involvement during prenatal life in humans. Human embryo implantation and fetal development are energy-intensive processes requiring substantial amounts of ATP. Consequently, mitochondrial dysfunction during these phases can compromise normal fetal development with an earlier manifestation before birth. Examples of metabolic stressors include environmental factors (i.e. diet, lifestyle, infections, toxic agents), or specific genetic conditions that may cause mitochondrial dysfunctions, the latter referred to as mitochondrial diseases (MDs).

This manuscript will review the evidence indicating that MDs can affect the developmental process during the fetal stages, in turn impairing fetal or pregnancy health, pointing out the fetal and maternal outcomes that could represent a wake-up call for targeted genetic diagnosis.

### Mitochondrial diseases

Mitochondrial diseases (MDs) are a group of genetic disorders caused by mutations in genes encoded by nuclear DNA (nDNA) and mitochondrial DNA (mtDNA), whose functions are necessary to sustain oxidative phosphorylation (OXPHOS), the final step of aerobic metabolism. Although considered rare individually, altogether, MDs are among the most prevalent group of inherited diseases, with a prevalence of approximately 1 in 4300 (22.9 in 100,000) in adults [6]. Since mitochondria are present in all nucleated cell types, MDs manifest as heterogeneous multisystem diseases occurring at any age and in any organ or tissue but primarily affecting those dependent on OXPHOS, such as the brain, skeletal muscle, heart, and eye [6, 7].

Due to dual genetic control (nuclear or mitochondrial), MDs can be classified into two principal categories, depending on which genome carries the responsible mutation and the inheritance pattern. Nuclear DNA mutations can follow an autosomal dominant (AD), autosomal recessive (AR) or X-linked pattern of inheritance, while pathogenic mtDNA mutations are strictly maternally transmitted, but sporadic mtDNA mutations can also occur [8]. Mutations in the same gene can result in dissimilar clinical presentations; for instance, mutations in the mitochondrially encoded MT-ATP6 gene clinically arise in different pathologies, including mitochondrial encephalomyopathy, cerebellar ataxia, severe kidney disease, and diabetes [9], neurogenic weakness, ataxia and retinitis pigmentosa (NARP) [10], and Charcot–Marie–Tooth disease [11]. In contrast, the genetic aetiology of a specific syndrome can be due to mutations in different genes, as in the case of Leigh syndrome (LS), which can be caused by nDNA and mtDNA mutations in more than 100 genes overall [12]. When mutations occur in the mitochondrial genome, the heteroplasmy level (i.e., the proportion of mutated and wild-type mtDNA) critically determines the manifestation of the OXPHOS defect [13].

The clinical manifestations of MDs range from the earliest stages of the neonatal period to the later stages of adulthood [14]. The natural history of the disease can be highly heterogeneous, from a slowly progressive syndrome punctuated by periods of good health [15], to subacute, rapidly progressive, and fatal neurological deficits in adulthood, as in the case of TTC19 mutations [16], to different phenotypes as for Thymidine kinase 2 deficiency syndrome [17].

Rearrangements (single deletions or duplications) of mtDNA are responsible for sporadic progressive external ophthalmoplegia (PEO), Kearns–Sayre syndrome (KSS) [18] and Pearson’s syndrome [19, 20]. A high degree of clinical and biochemical variability is also observed in the presence of homoplasmic mutations, such as in Leber’s hereditary optic neuropathy (LHON). The non-synonymous mutations at positions 11,778 in ND4, 3,460 in ND1, and 14,484 in ND6 account for 90% of cases. The disease is marked by incomplete penetrance, is more common in males, and often involves a spontaneous partial recovery of visual acuity. Additionally, environmental factors and polymorphisms in mtDNA haplogroups J1c and J2c are associated with increased disease penetrance [21, 22].

This extreme clinical, biochemical and genetic variability represent a severe obstacle, hindering the ability to gather homogeneous and sufficiently large patient cohorts, provide unequivocal evidence of specific clinical signs that facilitate a rapid diagnosis, and evaluate the efficacy of new treatments.

### Postnatal diagnosis of MDs

Due to the intricate interplay between mitochondrial DNA (mtDNA) and nuclear DNA (nDNA), as well as the clinical heterogeneity observed in MDs, molecular diagnosis is particularly complex. Nonetheless, accurate diagnosis is crucial for patients and their families as it significantly influences prognosis, treatment decisions, genetic counselling, and reproductive options. In response to this need, expert mitochondrial medicine laboratories in the UK have developed guidelines for genetic testing of mitochondrial disorders. However, these guidelines currently serve as recommendations and are applicable only within the UK.

A specific algorithm for genetic testing is detailed in a comprehensive review [23], outlining the latest strategies for the molecular diagnosis of mitochondrial diseases. In Europe, the initiative by the interERN working group on mitochondria seeks to establish standardized treatments for MDs [24] and certain specific manifestations, such as epilepsy [25]. Molecular diagnosis is essential for assessing reproductive options and prenatal diagnosis. Genetic counselling for MDs caused by nuclear genetic defects follows the patterns of other Mendelian inherited diseases, depending on the mode of inheritance (autosomal dominant, autosomal recessive, or X-linked). In contrast, genetic counselling for pathogenic variants of mtDNA is straightforward for males, as they do not transmit the variants to their offspring. However, genetic counselling for females is more complex due to the intricate transmission dynamics of mtDNA variants. The counselling process varies based on the pathogenic variant, its heteroplasmy level, and the mtDNA bottleneck effect, making disease risk prediction highly challenging.

Guidelines for reproductive options in families with MDs are discussed in detail elsewhere [26].

### Therapies for MDs

The genetic and biochemical complexity of MDs often presents a diagnostic challenge and impedes the development of effective therapies. This complexity also limits the ability to recruit large, well-characterized cohorts of patients for clinical trials. However, recent advancements in preclinical research offer promising prospects, and significant progress is anticipated in the coming decades.

Current proposed treatments were classified into two categories: (i) “one-size-fits-all” strategies, which can potentially be used to treat various MDs regardless of the underlying genetic mutation, and (ii) “precision medicine” approaches, aimed at treating a specific MDs with a particular mutation or distinct metabolic characteristic. The ‘one-size-fits-all’ strategies include symptomatic interventions such as diet, exercise, exposure to hypoxia, and pharmacological therapy. These therapies aim to (i) induce mitochondrial biogenesis, (ii) stimulate the nitric oxide synthase pathway, (iii) increase ATP synthesis, (iv) improve antioxidant defence, (v) enhance mitochondrial quality control by promoting fission/fusion events and autophagy of damaged mitochondria, and (vi) target cardiolipin. Recent advances in understanding the pathophysiology of several MDs have enabled the development of “precision medicine” approaches. Specialized therapies in this category include (i) nucleotide supplementation, (ii) replacing defective mtDNA in oocytes, (iii) supplementation of exogenous mitochondria, (iv) gene and cell replacement therapies, (v) scavenging of toxic metabolites, (vi) organ transplantation, and (vii) mtDNA editing. However, a comprehensive description of therapeutic strategies for MDs is beyond the scope of this manuscript; please refer to [27] for an exhaustive list.

## The role of Mitochondria in embryo-fetal development

### General considerations

Embryonic and fetal development encompasses the entire intrauterine differentiation, growth, and maturation process from conception to birth, during which nutrient, oxygen, and hormone levels fluctuate. Metabolic perturbation during these phases can affect fetal growth and tissue maturation, compromising postnatal organ function and the newborn’s health [28–30].

In the first stages of human fetal development, the embryo grows in a low-oxygen environment, with oxygen levels around 18,2 mm Hg in the amniotic fluid between weeks 11 and 16 of gestation [31]. At this stage, hypoxia plays a significant physiological role in fetal development, as it is involved in various processes such as placentation, angiogenesis and hematopoiesis [32]. The initiation of blood circulation in the placenta supplies oxygen to the developing embryo, as evidenced by a positive correlation between mean PO2 within the intervillous space and the gestational age [32]. Human fetal tissues from 9 to 17 weeks of gestation display fully assembled and enzymatically functional OXPHOS enzymes. However, biochemical activities are significantly lower than those detected in postnatal tissues [33].

After birth, when oxygen pressure rises significantly to about 26 mm Hg, there is an increase in mitochondrial biogenesis and activation of both nuclear and mitochondrial gene expression to cope with the high energetic requirements [34–36]. A significant coordinated increase in mRNA expression of genes encoding subunits of cytochrome C oxidase occurred from the neonatal stage to adulthood in various tissues of mice, including brain, skeletal muscle, and kidney [35]; a similar trend has been demonstrated for other OXPHOS complexes, including complex V [36]. Similarly, OXPHOS activities increase significantly after birth in several human tissues (e.g., liver, heart and muscle) compared to their fetal counterparts [33].

The mitochondria of the placenta play a crucial role in maintaining pregnancy. The placenta consists of two adjacent cell layers (cytotrophoblast and syncytiotrophoblast) that arise from the blastocyst and have very different mitochondrial and physiological functions. Cytothrophoblasts have typical organelle organisation, while syncytiotrophoblast mitochondria are small, spherical, and have a poor dense matrix with reduced complex V dimers, which shape mitochondrial cristae. The syncytiotrophoblast forms the outer layer of the placenta that directly contacts the maternal structures, and it is the placental region that is most sensitive to changes in oxygen levels, possibly due to its lower antioxidant capacity compared to cytotrophoblast mitochondria [37]. Reactive oxygen species (ROS) physiologically regulate many aspects of cell biology, including intracellular signaling and tissue adaptation. However, an excess of ROS can be harmful and lead to pregnancy complications like gestational diabetes mellitus (GDM) and pre-eclampsia [37]. The latter can also be caused by alteration in mitochondrial dynamics, although the precise relationships remain to be understood [37]. Like in other tissues, placental mitochondria respond to different metabolic insults and external stimuli: malnutrition, hypercholesterolemia, and obesity have all been associated with changes in placental mitochondrial mass, indicating metabolic plasticity [37].

The placenta performs nutritional and endocrine functions [38]. It optimises fetal growth by modifying the maternal supply of nutrients and oxygen. Glucose is the principal energy source for both the placenta and the fetus [29], and its supply is critically dependent on maternal circulation, as fetal gluconeogenesis is minimal.

Moreover, mitochondria drive oocyte maturation: in this process, the number of mitochondria and the mitochondrial genome gradually increase, the latter reaching ~ 250.000 copies *per* cell, significantly more than in other cell types with high energy requirements, such as muscle cells and neurons [39]. Interestingly, there is a close correlation between the number of mtDNA in oocytes and the fertilisation outcome, suggesting that mitochondria are instrumental in sustaining the initial stages of zygote growth [39, 40].

**Fig. 1:**
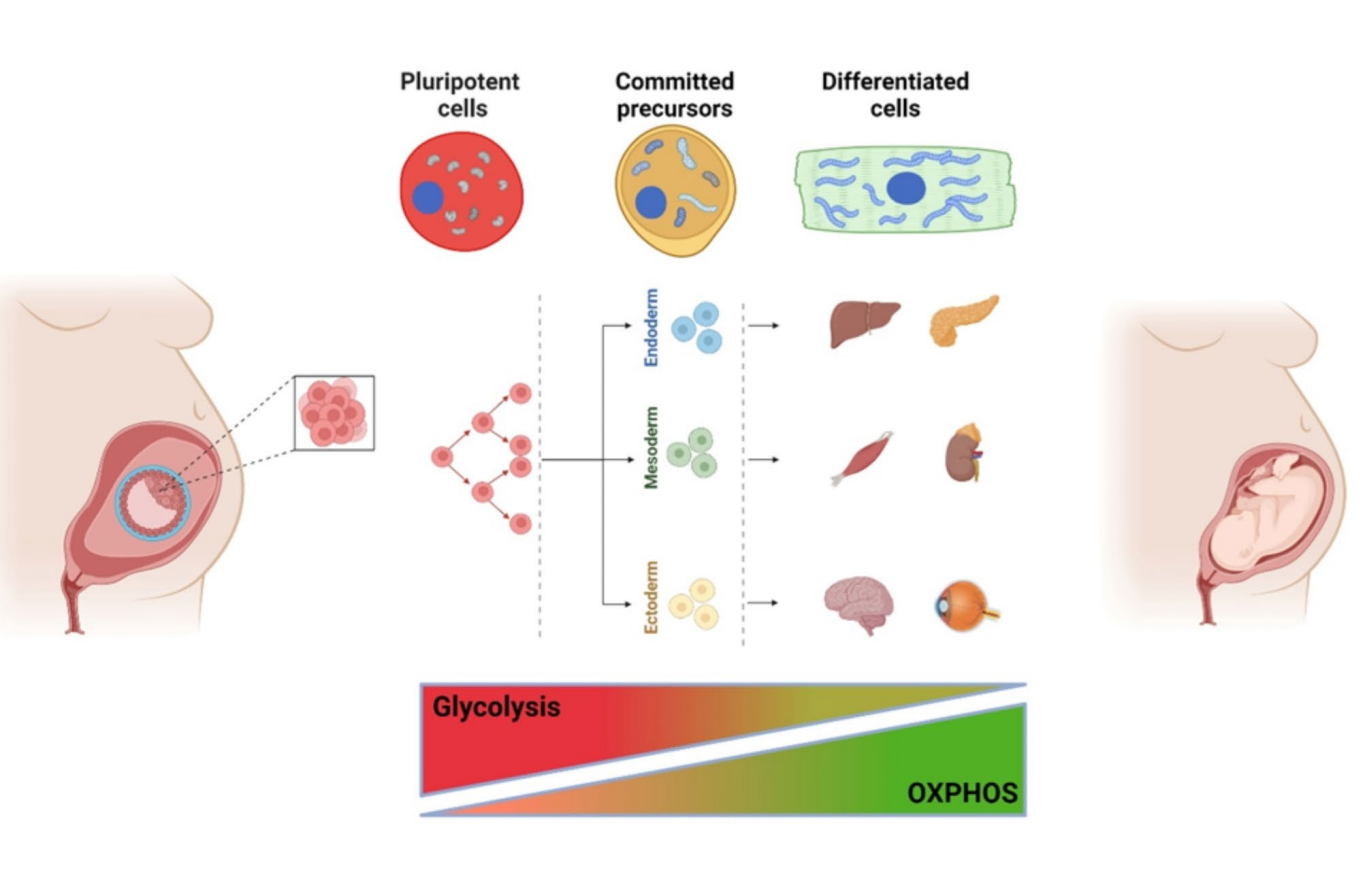
Schematic representation of the mitochondrial changes during the embryonic stem cell differentiation in somatic cells. Spherical-shaped mitochondria in pluripotent stem cells become elongated in differentiated cells. This process leads to changes in the cell metabolism that switch from glycolysis to OXPHOS

Mitochondria also play a pivotal role in the lineage commitment of cells (Fig. 1). Stem cells have the dual capacity to self-renew or commit towards a specific cell type. The balance between self-renewal and clonal expansion/differentiation, crucial for regulating stem cell populations’ size, entails different metabolic requirements. These processes are mainly studied in neural stem cells (NSCs) development [1, 3, 41–44] and cardiomyocyte maturation [28, 45]. Proliferating NSCs exhibit a glycolytic phenotype, which is necessary to support the synthesis of cell building blocks to maintain cell growth. Conversely, this metabolic asset switches to OXPHOS during differentiation to generate ATP more efficiently [1, 43]. The mitochondrial cristae maturation highlights the metabolic switch occurring during cell lineage commitment, and a parallel increase in mtDNA copy number accompanies it. Undifferentiated stem cells maintain a low mtDNA pool to preserve their stemness and high proliferation potential. Committed cells increase their mtDNA molecules up to a cell-specific amount in the final differentiated state, as seen in several in vitro and in vivo models [41, 42, 46, 47].

Similarly, mitochondria mediate the molecular processes that trigger the maturation of cardiomyocytes. During the perinatal window, mitochondria transit from tiny, fragmented organelles to an extensive network with developed cristae with high oxidative capacity. Such organization supports the contractility demand of the postnatal heart, in which the cardiomyocytes have a high density of mitochondria tightly packed between the sarcomeres [28, 45].

### The impact of MDs in embryo-fetal development – preclinical evidence

**Fig. 2:**
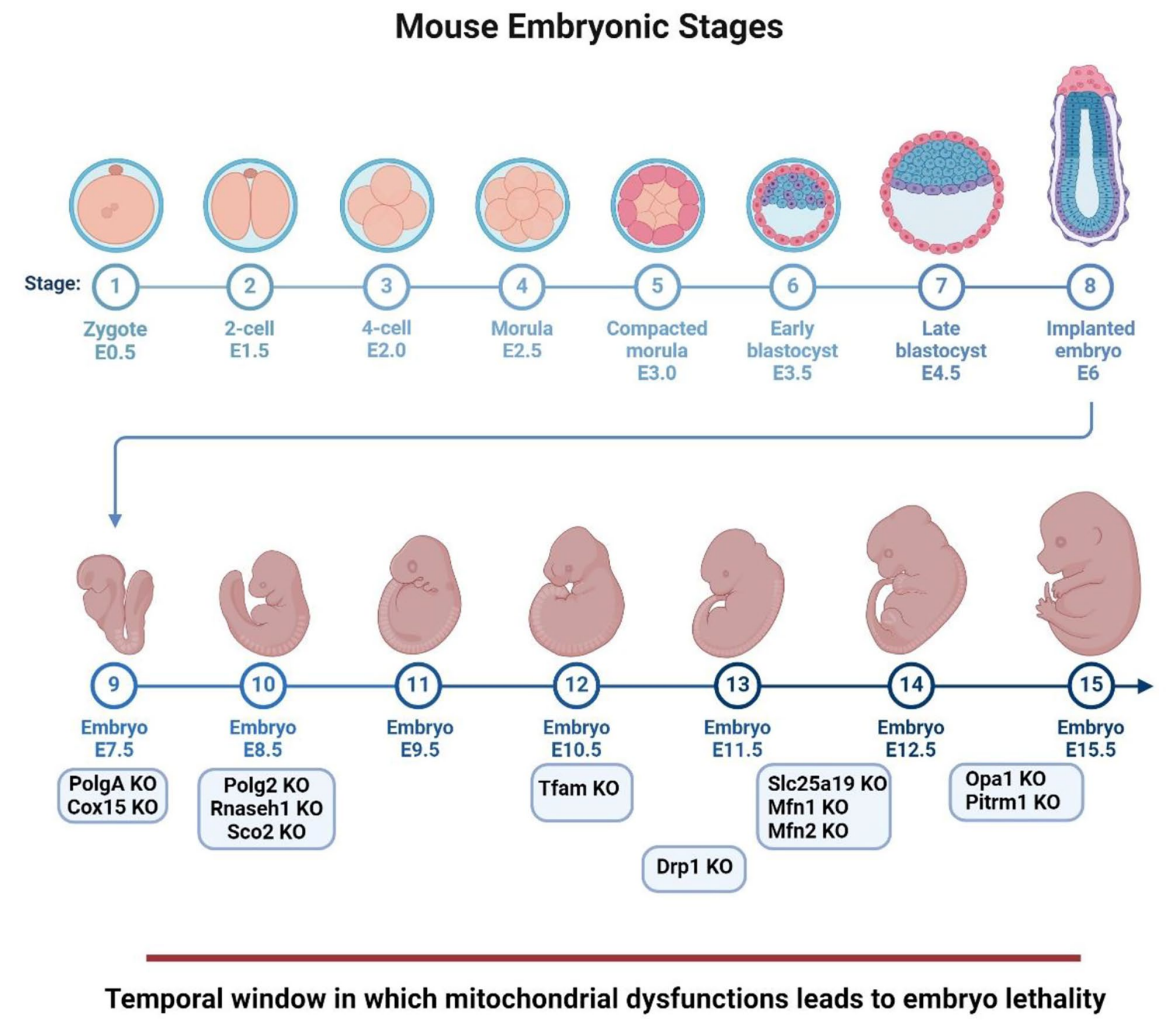
Embryonic lethality in different MD mouse models usually occurs between E7.5 and E13.5. Adapted from “Mouse Development Embryonic Stages” assembled using dynamic BioRender assets. Retrieved from https://app.biorender.com/biorender-templates/figures/all/t-6009a97e28bf69021fdce6e1-mouse-development-embryonic-stages

Over the past twenty years, numerous mouse models of MDs have been generated. Many of these have shown that the constitutive removal of a gene required for mitochondrial OXPHOS is incompatible with life, causing embryo lethality or generating developmental disorders (Fig. 2). Below, we report the findings obtained by different in vitro and in vivo genetic models with altered embryonic development and categorize them by type of mitochondrial dysfunction. A summary is reported in Table 1

**Table 1:** Preclinical manifestation of mitochondrial disease in embryonic development

*Replication And Maintenance Of Mitochondrial Genome*	Gene	Model	Preclinical Manifestations	Ref
	*Tfam*	Constitutive knockout mouse	Embryonic lethality between E8.5 and E10.5 Smaller size Delayed neuronal development Absence of optic disc and cardiac structure Indistinct somites Nearly complete absence of mtDNA Enlarged mitochondria with disorganised cristae	[50]
	*PolgA*	Constitutive knockout mouse	Embryonic lethality within day E7.5 and E8.5 No COX activity Barely detectable levels of mtDNA	[51]
	*Polg2*	Constitutive knockout mouse	Embryonic lethality within day E8.5 Loss of mtDNA and mtDNA gene products Ultra-structural defects Loss of organized cristae Accumulation of lipid droplets in embryonic tissue	[52]
	*Rnaseh1*	Constitutive knockout mouse	Embryonic lethality within day E(8.5) Reduction of mtDNA copy number at E7.5 mtDNA depletion Developmental arrest	[54]
	*Mpv17*	Constitutive knockout zebrafish	Developmental defects in muscles, liver, and energy supply Abnormal growth of the larvae during the developmental stage	[57]
*OXPHOS and Bioenergetic Metabolism*	*Ndufs4*	Constitutive knockout mouse	Low developmental rate of knockout embryos from knockout gametes	[62]
	*Ndufs2*	hGFAP-Cre-NDUFS2 knockout mouse	Decrease in cortical thickness and subtle hippocampal abnormalities at P0	[63]
	*Sdhd*	hGFAP-Cre-SDHD knockout mouse	Dilation of ventricles at P0 Marked atrophy of the cerebral cortex Virtual absence of the hippocampus and cerebellum	[64]
	*Surf1*	Constitutive knockout mouse	Embryonic lethality at gastrulation (E4–E7), organogenesis (E8.5–E12), and during body-mass growth and organ maturation (E13–E18)	[65]
		Constitutive knockout mouse	Lack of any prenatal phenotype	[66]
		Constitutive knockout pig	Significant reduction in cerebral cortex gray matter thickness, and disorganised cortical structure with several immature neurons at P0	[67]
		Human Neural precursor cells harbouring c.530T > G and c.769G > A homozygous mutations	Metabolic impairment which prevents correct neuromorphogenesis	[44]
		Human brain organoids harbouring c.530T > G and c.769G > A homozygous mutations	Loss of neurogenic progenitor zones	[44]
	*Sco2*	Constitutive knockout mouse	Embryonic lethality within E8.5 Severe morphological abnormalities Profound CIV deficiency	[68]
	*Cox15*	Constitutive knockout mouse	Embryonic lethality at E7.5	[69]
	*DLD*	Human iPSCs harbouring c.100 A > G mutation	Upon neural induction, reduced neural rosettes and neural rosette lumen area; Defects in cortical layer formation; Dysregulation of neuronal and glial markers	[70]
	*PDHA1*	Human iPSCs with c.79delC	Upon neural induction, reduced neural rosette lumen area; Defects in cortical layer formation; Dysregulation of neuronal and glial markers	[70]
	*AIF*	AIF-Foxg1Cre knockout mouse	Thinning of the cortex and enlargement of the ventricle in embryonic brain at E15.5 Reduced self-renewal capacity of NSCs in vitro Dysregulation of NPCs proliferation at E12.5	[71]
		AIF-Emx1-Cre knockout mouse	Dysregulation in NSCs fate decisions: decrease in symmetric self-renewing divisions and increased progenitor proliferation at E15.5 Cortical thinning and enlarged ventricles at the end of neurogenesis (E18.5)	[71]
	*Slc25a19*	Constitutive knockout mouse	Neural-tube closure defect and a yolk sac erythropoietic failure at E10.5 100% prenatal lethality by E12	[72]
*Genes Involved in Mitochondrial Dynamics*	*Drp1*	Constitutive knockout mouse	Embryonic lethality between E10.5 and E12.5 Smaller body size, less developed heart, poorly developed liver and thinner neural tube cell layer at E9.5–11.5	[77]
		Drp1-Nestin-Cre knockout mouse	At E18.5: Reduced size of the forebrain with expanded subdural space and ventricles Hypoplasia of white matter Periventricular leukomalacia Disintegration of the deepest cortical layer	[77]
		Drp1 shRNA at E13.5 mouse	Decreased proportion of generated neurons and increased proportion of intermediate progenitors and RGCs	[78]
	*Mfn1*	Constitutive knockout mouse	Embryonic lethality before E11.5–12.5 Pronounced developmental delay	[79]
	*Mfn2*	Constitutive knockout mouse	Embryonic lethality before E11.5–12.5 Severe disruption of the placental trophoblast giant cell layer	[79]
		Meox2-Cre-Mfn2 knockout mouse	Severe abnormalities al P0 Pronounced mortality at P1 Reduction in the size of the cerebellum	[80]
	*Opa1*	Opa1 (Q285STOP) mouse	Embryonic lethality at E13.5 Small size and gross morphological abnormalities at E11.5	[81]
*Genes involved in mitochondrial proteostasis*	*Pitrm1*	Constitutive knockout mouse	Embryonic lethality at E13.5	[83]
	*Clpp*	Constitutive knockout mouse	Defective oocyte maturation and embryo development	[84]

#### Genes involved in the replication and maintenance of mitochondrial genome

Compared to the nuclear genome, relatively few proteins are involved in the replication, maintenance and repair of the mitochondrial genome. The DNA polymerase γ (Polγ) is the only enzyme synthesising and repairing mtDNA. This enzyme possesses two distinct subunits in animal cells: the catalytic subunits Polymerase γ A of 140 kDa (encoded by the POLGA gene) and the Polymerase γ 2 of 55 kDa (encoded by POLG2), which has DNA polymerase, 3’-5’ exonuclease, and 5’dRP lyase activities [48]. Other known gene products involved in mtDNA replication, transcription, and stability include (i) the mitochondrial transcription factor A (mtTFA, encoded by the TFAM gene), which is involved in mtDNA replication; (ii) the mitochondrial single-strand binding protein (mtSSB), which enhances helix destabilisation, and regulates mtDNA replication initiation [49]; (iii) the mitochondrial helicase, Twinkle, an essential helicase that unwinds the mitochondrial duplex genome during DNA replication [48].

Different studies reported that the depletion of genes encoding for the mtDNA replication machinery halts embryonic development. Larson and colleagues demonstrated that disruption of *Tfam* leads to severe mtDNA depletion in vivo with abolished oxidative phosphorylation. The knockout embryos undergo implantation and gastrulation but die before embryonic day (E)10.5, demonstrating the fundamental role of *Tfam* in mitochondrial biogenesis and embryonic development [50]. Similarly, the constitutive knockout of the *PolgA* gene in mice resulted in embryonic death at E7.5 [51], while the absence of *Polg2* resulted in death at E8-8.5 [52]. Deletion of the ribonuclease gene *Rnaseh1*, encoding for an endonuclease present in both the nucleus and mitochondria that digests the RNA component of RNA-DNA hybrids [53], led to embryonic developmental arrest at E8.5 [54]. This failure of embryo survival was linked to a reduction of mtDNA copy number at E7.5, while total chromosome content remained consistent across the genotypes [54]. Recently, a novel zebrafish model knockout for mpv17 – encoding for an inner mitochondrial membrane channel involved in translocating key metabolites [55], whose mutations in humans are associated with a severe mtDNA depletion syndrome [56] - showed altered embryonic development, with delayed and abnormal liver and skeletal muscle development in larvae, reduced energy supply, and compromised mitochondrial ultrastructures [57]. Overall, these data suggest that mtDNA is required to maintain embryonic development during gastrulation and organogenesis [58], and that failure of this process leads to severe mtDNA depletion and developmental arrest.

#### Genes involved in OXPHOS and Bioenergetic Metabolism

Pre-implantation embryonic development involves the transition from zygote division to blastocyst formation. Mitochondrial oxidative metabolism is a significant contributor of ATP during the entire preimplantation stage, with over 85% of all ATP produced in the mouse blastocyst derived from mitochondria [59]. Insufficient ATP levels are associated with impaired spindle function in mouse metaphase II oocytes [60] and lower quality of the resulting embryos [59].

Transcriptome profiling data revealed significant differences in the expression of numerous mitochondria-related genes between embryos conceived by in vitro fertilisation (IVF) and those conceived by in vivo fertilisation (IVO) [61], including genes encoding for electron transport chain subunits, β-oxidation genes, mitochondrial biogenesis genes, and glutathione metabolism [61]. These effects could be significantly associated with compromised development of IVF embryos.

A clear demonstration of the crucial role of mitochondrial complex I (CI) in embryo development comes from the characterization of the constitutive *Ndufs4* knockout (KO) mouse model of Leigh syndrome (LS). This study revealed that the developmental progression of zygotes derived from *Ndufs4* KO sperms and *Ndufs4* KO oocytes was markedly lower compared to wild-type (WT) embryos at various stages: 2-cell stage (78.4% vs. 97.5%), 4-cell stage (62.2% vs. 92.5%), morula stage (51.4% vs. 85%), and blastocyst stage (29.7% vs. 70%). [62]. These data highlight the importance of preserved bioenergetic function in efficient embryonic development.

Cabello-Rivera et al. explored the effects of dysfunctional CI on NSCs and radial glial cells (RGCs), a cell type originating from the primordial neuroepithelium, which serves as a stem cell niche for generating neurons, astrocytes and oligodendrocytes. They created a conditional knockout model hGFAP-NDUFS2 in which the expression of the complex I subunit NDUFS2 is suppressed in cells expressing the Cre recombinase under the control of human glial fibrillary acidic protein (hGFAP) promoter. CI ablation in NSCs resulted in early perinatal mortality and compromised the postnatal development of the dorsal cortex, corpus callosum, hippocampus, and cerebellum [63]. Similarly, the hGFAP-SDHD mouse, with conditional ablation of the membrane-anchoring subunit D of succinate dehydrogenase [64], had a profound alteration of neuronal maturation during brain development, which caused brain atrophy and early mortality [64].

Again, mitochondrial dysfunctions caused by complex IV (CIV) defects are associated with altered embryonic development. The first constitutive mouse model with disrupted SURF1, a CIV assembly factor associated with LS, showed high post-implantation embryonic lethality, affecting approximately 90% of the *Surf1* knockout individuals [65]. Although the prenatal manifestation of *Surf1* ablation was not observed in the second knockout mouse model [66], other recent findings further suggest that mitochondrial dysfunction in SURF1-associated Leigh syndrome probably begins at an early stage of development. Newborn SURF1 knockout piglets had a significant reduction in cerebral cortex gray matter thickness and a disorganised cortical structure with several immature neurons, indicative of a delay in central nervous system development [67]. In addition, embryonic lethality during early gestation was also observed in the *Sco2* knockout mouse, a model recapitulating a fatal infantile cardio-encephalomyopathy with CIV deficiency. Knockout embryos had severe morphological abnormalities, profound CIV deficiency and could not survive more than E8.5 [68]. Also, mice knockout for *Cox15* gene - involved in the biosynthetic pathway of heme a, the CIV-specific heme moiety - showed consistent embryonic lethality at E7.5 [69]. Human neural progenitor cells (NPCs) derived from LS patients with *SURF1* mutations had significant metabolic impairment, being unable to switch from glycolytic to OXPHOS metabolism, impairing neuronal morphogenesis [44]. These defects were observed in patient-derived 2D neuronal cells and in 3D cerebral organoids, which displayed a loss of neurogenic progenitor zones that formed around cavities resembling embryonic ventricles in healthy organoids [44]. Again, induced pluripotent stem cells (iPSCs) lines derived from LS patients carrying mutations in pyruvate dehydrogenase complex (PDHc) E1 alpha 1 subunit (PDHA1) and dihydrolipoyl dehydrogenase (DLD) genes showed several abnormalities in three-dimensional models of brain development, with pronounced defects in neural epithelial bud generation, size and cortical architecture, suggesting prenatal impairment [70].

Khacho et al. investigated the consequences of mitochondrial dysfunction during cortical development using a model with deleted mitochondrial oxidoreductase protein AIF, located in the inner mitochondrial membrane, whose deficiency leads to impaired mitochondrial respiration, reduced CI and CIII functions and increased production of ROS [71]. Genetic ablation of the AIF gene in uncommitted cells of the early telencephalon at E9 - before the onset of neurogenesis - caused severe mitochondrial dysfunction in mice and impacted embryonic neurogenesis and the correct brain development. NSCs self-renewal, the proliferation of neural progenitor cells, their exit from the cell cycle, and the process of neuronal differentiation were all affected by AIF deletion [71].

Another example of mitochondrial dysfunction leading to impaired embryonic development is represented by mutations in SLC25A19, a nuclear gene encoding for the mitochondrial thiamine pyrophosphate (TPP) transporter. Mutations in SLC25A19 are associated with Amish lethal microcephaly (MCPHA), a condition characterised by severely impaired brain development and α-ketoglutaric aciduria [72]. The *Slc25a19* knockout mouse model presents 100% prenatal lethality within E12. Affected embryos at E10.5 have a neural-tube closure defect with a yolk sac erythropoietic failure, ruffling of the neural fold ridges, and elevated α-ketoglutarate in the amniotic fluid [72].

#### Genes involved in mitochondrial dynamics

The intracellular mitochondrial morphology and distribution switch dynamically in response to the cell’s metabolic state. A remarkable change in mitochondrial fission and fusion has been observed in early development [73–75]. To investigate the prenatal role of mitochondrial fission, Ishiara and coworkers generated *Drp1*-knockout mice deleting the exon 2, encoding the GTP-binding motif. Constitutive ablation of *Drp1* resulted in embryonic lethality between E10.5 and E12.5. At E9.5–11.5, the knockout embryos had a significantly smaller body size, a pulsating - but less developed - heart, a poorly developed liver and a thinner neural tube cell layer [76, 77].

Additionally, neuron-specific conditional knockout (NS-*Drp1* KO) mice died shortly after birth. Knockout embryos analyzed at E18.5 were similar in size to wild-type counterparts and showed no gross abnormalities. However, histological analysis revealed a reduction in the size of the forebrain with enlarged subdural space and ventricles, mainly due to white matter hypoplasia [77].

Similarly, Iwata and colleagues suppressed Drp1 expression by *in utero* electroporation using short hairpin RNA at E13.5 and observed a decrease in the proportion of generated neurons and an increase in the proportion of intermediate progenitors and RGCs [78]. Through an elegant approach based on *in utero* labelling of the mitochondrial network, they demonstrated that mitochondrial dynamics after mitosis influence mouse cortical neurogenesis [78].

Further studies highlighted the crucial role of mitochondrial fusion during fetal growth. Mice lacking *Mfn1* or *Mfn2* exhibited embryo lethality before E11.5–12.5 [79]. *Mfn2* knockout embryos presented a specific and severe disruption of the placental trophoblast giant cell layer, which was not observed in *Mfn1* knockout embryos. *Mfn1* and *Mfn2* knockout mice did not survive mid-pregnancy [79]. Since embryonic lethality precluded the investigation of the role of mitochondrial fusion in later development, the authors created a conditionally inactivated allele of *Mfn1* and *Mfn2*. The successful generation of Meox2-Cre/Mfn2^loxP^ mice [80], in which *Mfn2* was ablated throughout the embryo, but not in cell lineages giving rise to the placenta, confirmed that placental insufficiency underlies the embryonic lethality of the previously described [79] Mfn2 knockout mice. However, Meox2-Cre/Mfn2^loxP^ mice exhibited dramatic abnormalities and several died at P1 [80]. Additionally, *Mfn2*, but not *Mfn1* was required to develop and maintain the cerebellum properly [80].

Similar embryo lethality affected the *Opa1* constitutive knockout mouse model at E13.5. Homozygotes were detected at E11, although at this stage, they were significantly smaller than controls and displayed growth retardation and gross morphological abnormalities [81].

#### Genes involved in mitochondrial proteostasis

Preclinical evidence also points to perturbations in mitochondrial proteostasis that may affect embryonic development. One example is Pitrilysin metallopeptidase 1 (PITRM1), an enzyme located in the mitochondrial matrix in charge of digesting oligopeptides, including mitochondrial target sequences that are cleaved from proteins imported across the inner mitochondrial membrane [82]. Biallelic pathogenic variants of *PITRM1* are associated with a slowly progressive syndrome characterised by intellectual disability, spinocerebellar ataxia, cognitive decline and psychosis. The constitutive *Pitrm1* knockout mouse showed embryonic lethality at E13.5 [83], pointing to the role of mitochondrial quality control in proper embryonic development.

An intriguing link between dysfunctional mitochondrial proteostasis and fertility is observed in CLPP-associated Perrault syndrome. CLPP gene encodes the proteolytic subunit of caseinolytic mitochondrial matrix peptidase [84], and its mutations in patients lead to sensorineural hearing loss and ovarian dysfunction, which can range from gonadal dysgenesis - presenting as primary amenorrhea - to primary ovarian insufficiency [85]. The role of *Clpp* in oocyte quality was recently confirmed by analysing the ovarian phenotype in a mouse model. This analysis confirmed abnormalities in oocyte mitochondrial function [84]. A significant underrepresentation of *Clpp* KO pups born from heterozygous matings indicated a reduced intrauterine survival due to the absence of CLPP.

## Clinical manifestations of mitochondrial disease in pregnancy

In this section, we review (i) the impact of pregnancy on patients affected by MDs, and (ii) the effect of MD-affected fetuses on maternal health.

**Fig. 3:**
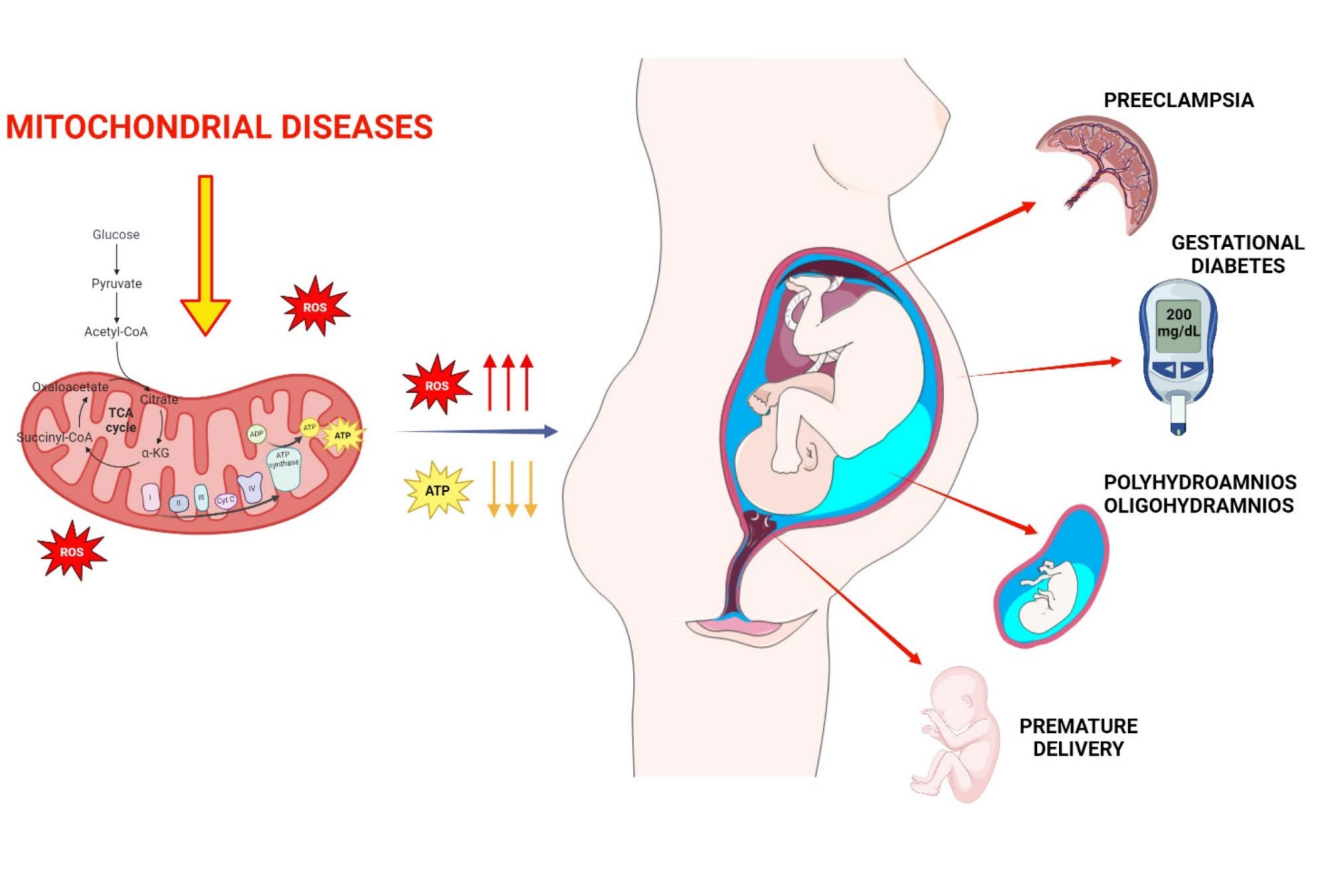
Reported pregnancy complications in women affected by MDS: pre-eclampsia, gestational diabetes, polyhydramnios, oligohydramnios, and premature delivery. See Sect. 3 for details. Created with BioRender.com

Pregnancy places a significant metabolic demand on the body, necessitating efficient energy production to support both physiological changes and fetal growth. Women affected by MD, who already have impaired mitochondrial activity, may not be able to sustain the additional energy request and may experience increased pregnancy complications. The most common pregnancy-related conditions include Pre-Eclampsia (PE), Gestational Diabetes (GDM), Polyhydramnios, Oligohydramnios and Preterm Birth (PB) (Fig. 3). Likewise, subclinical MDs in women can exacerbate and manifest during pregnancy, often remaining misdiagnosed [86]. MELAS is the most common MD whose diagnosis comes for the first time during pregnancy. Annaiah et al. described a woman - carrying the most common mtDNA mutation m.3243 A > G in the tRNA^Leu^ gene - who experienced complications in pregnancy, including spontaneous abortion, GDM, premature rupture of membranes and intrauterine death. Post-operative examination revealed hypertension, pulmonary embolism and cardiomyopathy as a complication of MELAS [87]. In another case, Moriarty et al. described a pregnant woman at 38 + 5 weeks of gestation carrying the m.3243 A > G mutation and suffering from PE and toxicity to magnesium sulphate [86].

Furthermore, the risk of maternal complications during pregnancy and labour appears to be increased in cases of previously identified maternal MD. In 1998, Yanagawa et al. described the case of a 31-year-old woman earlier diagnosed with a MELAS-like syndrome, admitted at 27 weeks of gestation because of muscle weakness and paresthesia in the distal extremities. She developed tachycardia, loss of muscle strength without the ability to stand on her own, and metabolic acidosis, so myopathy and axonal neuropathy were diagnosed as atypical manifestations of the syndrome. The symptoms gradually improved without pharmacological treatment, and both the birth and the postnatal periods were uneventful. In this case report, the authors detected the same mtDNA mutation in the umbilical cord sample as in the patient’s blood [88] The following year, Kovilam et al. reported the case of a pregnant 29-year-old woman with a previous diagnosis of MELAS who developed severe migraine-like events, flaccid and proximal weakness due to demyelinating polyneuropathy, expressive aphasia, bilateral retinitis pigmentosa, sensorineural hearing loss, cardiac arrhythmias, dumping syndrome, and pelvic floor relaxation with cystocele. Pregnancy complications included GDM and Fetal Growth Restriction (FGR, *see Sect. 4*). In this patient, labour was induced at 36 weeks’ gestation due to deterioration of the condition [89]. A review by Say et al. [90] described the risk of women with MD developing complications during pregnancy. Most of these women carried the MELAS mutation, while others had only a biochemical diagnosis without a molecular genetic definition. The most commonly reported complications were preterm labour and pre-eclampsia, although the complete absence of complications was also reported. A summary of the clinical manifestations of MDs in pregnancy is reported in Table 2.

**Table 2:** Clinical manifestation of mitochondrial disease in pregnancy

Clinical Study and Genetic Mutation(s)	Pregnancy complications	% in healthy controls	Ref
60 women carrying the m.3243 A > G; mean heteroplasmy % in UEC: 19.9% (range 5–85%).	Pre-eclampsia 12% Gestational diabetes 11% Premature delivery 25.3%	Not reported	[103]
A woman with m.3243 A > G (heteroplasmy level not reported)	Pre-eclampsia Threatened premature labor Magnesium intolerance	N.A.	[104]
103 women with MD or mitochondrial dysfunction (370 pregnancies)	Miscarriages (29.2%) Preterm labour (11.7%) Premature delivery (10.1%) Pre-eclampsia (5.2%) Gestational diabetes (6.8%)	From the literature review: Miscarriages (8–20%) Preterm labour (not reported) Premature delivery (not reported) Pre-eclampsia (2–3%) Gestational diabetes (1–25%)	[105]
Heteroplasmic carriers of different mtDNA mutations (*n* = 31)	Gestational diabetes 13.6% (*p* < 0.05); Miscarragies 26.3% (*p* < 0.05) FGR 10% (*p* < 0.05) Postpartum haemorrhage 12.2% (*p* < 0.01) Termination of pregnancy 20.1% (*p* < 0.0001) Live birth 51.8% (*p* < 0.001)	Gestational Diabetes: 3% Miscarriages 11.1% FGR 1% Postpartum haemorrhage 2% Termination of pregnancy 0.8% Live birth 87.2%	[113]
26 women with heteroplasmic m.3243 A > G (*n* = 25) or m.12,258 C > A (*n* = 1); mean heteroplasmic rate 18% (88 pregnancies)	Gestational diabetes (24%) Premature delivery (13.6%) Miscarriages (25%)	From Irish database www.irishhealth.com: Miscarriages (15%) Premature delivery (5%) Gestational diabetes: not reported	[114]
220 women carrying heteroplasmic variants m.4917 A > G m.4216T > G m.10398G > A	Premature delivery (median gestational age: 33 weeks; *p* < 0.01)	Median gestational age: 39 weeks	[120]
A woman with CPEO (2 pregnancies)	Preterm labour Hypertension	Not reported	[122]

### Pre-eclampsia

Pre-eclampsia (PE) is a complication that occurs in 2–4% of all pregnancies. Together with gestational hypertension and chronic hypertension, it belongs to the spectrum of hypertensive disorders of pregnancy (HDP). HDPs are the leading cause of maternal and perinatal morbidity and mortality, especially in early-onset and underserved areas. Hypertension in pregnancy is defined as systolic blood pressure > 140 mmHg and diastolic blood pressure > 90 mmHg based on an average of at least two measurements. PE is the most dangerous form of HDPs as it can occur suddenly and progress rapidly without any clear warning. The presence of *de novo* hypertension after 20 weeks’ gestation, evidence of proteinuria (> 30 mg/mmol urinary protein) and/or maternal acute kidney injury, liver dysfunction, neurological features, hemolysis/thrombocytopenia, or fetal growth restriction make the diagnosis of pre-eclampsia [91].

The initial process of placentation requires a low-tension oxygen environment to stimulate implantation and early invasion of the placenta through a specific oxygen-sensing signaling pathway linked to hypoxia-inducible factor (HIF-1), the primary regulator of transcription of genes in response to hypoxia [92].

As pregnancy progresses, placental maturation depends on establishing uteroplacental circulation and increasing energy and oxygen availability, all required for trophoblastic cell differentiation and fetal growth. Mitochondrial ATP production supports this shift from low-to-high oxygen tension and placental function during pregnancy by continuously increasing activity [93]. There is evidence that the third-trimester placenta contains more mitochondria than the first-trimester placenta, with the first-trimester mitochondria being more efficient at utilizing low oxygen levels [94].

In 1996 Ness and Roberts [95] postulated the existence of two types of PE: (i) an early-onset PE, which results in preterm birth before 34 weeks’ gestation and correlates with the so-called “placental insufficiency”, commonly associated with FGR, and (ii) late-onset PE (> 34 weeks), less frequently linked to FGR and attributed to maternal inflammatory conditions, including diabetes mellitus, chronic hypertension, obesity, and autoimmune disorders.

The etiopathogenesis of PE remains uncertain and multifactorial. However, it is widely accepted that PE must be responsible for premature placental impairment based on the following mechanisms: defective endovascular trophoblastic invasion with faulty spiral arteries’ remodelling and deregulation of anti-angiogenic factors production, inflammatory leukocytes (Natural Killer, NK) and ROS, causing widespread endothelial damage.

The involvement of mitochondrial dysfunction in preeclampsia was first reported in 1989 when Torbergsen et al. described a family with mitochondrial dysregulation and a high incidence of preeclampsia and eclampsia [96]. Since then, various scientific reports have provided evidence of impaired mitochondrial fusion/fission dynamics, lower activity of complex II of the electron transport chain, reduced expression of complexes I and IV, and mitochondrial swelling and broken cristae in placentas from pregnancies with preeclampsia compared to those from healthy pregnancies. As early as 1990, structural changes of mitochondria in the pre-eclamptic placenta were described, including an increase in size, cristae spacing, decreased MRC efficiency and oxygen consumption rate with reduced CIV [97–99]. Later on, Shi et al. demonstrated the dysregulation of 26 mitochondrial proteins in the pre-eclamptic placenta, which alter the processes of apoptosis, fatty acid oxidation, electron transport chain and oxidative stress [100].

Dysfunctional mitochondria produce high levels of ROS, which cause mitochondrial damage and, eventually, cell death. Damaged mitochondria release circulating mtDNA and peptides (also known as Damage-associated molecular patterns or DAMPs) that activate the immune system and initiate a cascade of events that determines the switch from non-cytotoxic NK to cytolytic NK in the area of spiral arteries, ultimately leading to a failure of vascular remodelling, a loss of vessel reactivity to vasodilator stimuli, and placental ischemia. Therefore, women diagnosed with PE have elevated levels of circulating mtDNA, indicative of mitochondrial damage.

A detailed explanation of pathomechanisms that link mitochondrial dysfunction to PE, was recently reviewed by Jahan et al. [101]. Please refer to this study for potential markers and prospective therapies.

As more markers of mitochondrial dysfunction emerge in the contribution of PE pathogenesis, new therapeutic strategies are being tested. Classic antihypertensive treatments may benefit from an adjuvating anti-inflammatory and antioxidant molecule. The most promising drugs and nutraceuticals considered are Resveratrol, Nicotinamide, MitoQ/mitoTEMPO and Metformin, which target mitochondrial pathways responsible for homeostasis and aim to reduce ROS production and sFLT1 circulating levels while maintaining OXPHOS function. Resveratrol has shown a significant contribution in previous randomized control trials as adjuvant treatment combined with nifedipine, resulting in more rapid and prolonged control of hypertension compared to nifedipine alone. It acts as a mitochondrial complex I-inhibitor, reducing ROS production and sFLT1 circulating levels. Administrations of metformin during pregnancy in cases of obesity, GDM and type 2 diabetes drive reduced sFLT1 and endoglin production by regulating mitochondrial function through oxidative phosphorylation, mnSOD and AMP kinase.

Furthermore, MitoQ/mitoTEMPO reduce mitochondrial ROS production by neutralizing free radicals or by a superoxide dismutase action. NAM decreases blood pressure and proteinuria, and prevents glomerular endotheliosis. It reduces HIF1-a expression and increases levels of NAM, NAD + and ATP [102].

Several studies highlighted a significantly higher rate of obstetric complications in women affected by MDs. Paul de Laat et al. described the prevalence of obstetric complications in a cohort of 85 female m.3243 A > G carriers compared to the Dutch population. Of the 65 women who completed follow-up, the prevalence of PE was 12% (20% in nulliparous and 63% in multiparous), with an odds ratio of 7.0 for nulliparous. The mean gestational duration of these pregnancies was 35 +/- 4 weeks, with no differences or correlation with the heteroplasmy level or Newcastle Mitochondrial Disease Scale for Adults (NMDAS) score [103].

Nakamura and colleagues reported the case of a 33-year-old, gravida 3, primiparous, pregnant woman with a history of 3 spontaneous abortions and the presence of type I diabetes mellitus, diagnosed at 18 years of age. Genetic analysis revealed the presence of a mtDNA point mutation m.3243. The patient developed PE at 33 weeks’ gestation, which led to an emergency cesarean Sect. [104]. In a recent retrospective study, Karaa and colleagues analysed 370 pregnancies reported by 103 respondents with MDs, of which 248 resulted in a live birth. PE was diagnosed in 18.4% of patients [105].

All these findings indicate that pregnancies complicated by MDs are associated with a higher risk of PE (12–18% prevalence in MDs *versus* 2–4% prevalence in the control population) and preterm birth, and should enrich the comprehension of the pathogenesis of PE while bridging the gap between mitochondrial dysfunction and the increased incidence of obstetric complications. Therefore, obstetricians must recognise MDs as a significant perinatal risk factor.

### Gestational diabetes

Gestational diabetes mellitus (GDM) is a pregnancy-associated disorder characterized by impaired glucose tolerance and vascular dysfunction in the fetal-placental unit. The global prevalence of GDM varies considerably, primarily due to the use of different diagnostic criteria. A 2019 meta-analysis, applying the International Association of Diabetes and Pregnancy Study Groups (IADPSG) criteria, reported the highest pooled prevalence of GDM in South Asia (Bangladesh, India, and Sri Lanka) at 11.4%, compared to 3.6–6.0% in other regions [106]. At the cellular level, diabetes mellitus (DM) is intricately linked to mitochondrial alterations, affecting both structure and function. The relationship between DM and mitochondrial dysfunction was first described by Yamada et al. in 1975 [107]. Since then, extensive research has highlighted a strong association between the progression of DM and mitochondrial impairment. Key features of mitochondrial dysfunction in DM include impaired mitochondrial biogenesis, altered ultrastructure, reduced activities of multienzyme complexes, decreased ATP production, and increased reactive oxygen species (ROS) production [108]. Mitochondria play an essential role in maintaining placental function. In GDM, as in type 1 and type 2 diabetes mellitus, mitochondrial dysfunction occurs in the placental endothelium and trophoblasts, leading to reduced oxidative phosphorylation and increased ROS production [109]. This emerging evidence suggests that mitochondrial dysfunction in the placenta may not only be a consequence but also a contributing factor to the development of GDM during pregnancy [37].

Numerous epidemiological studies have demonstrated that women who experience GDM have a heightened risk of developing T2DM later in life [110]. Indeed, GDM and T2DM share similar underlying pathophysiological mechanisms. Changes in mitochondrial function impact fatty acid metabolism, particularly triacylglycerol storage in adipocytes. Reduced mitochondrial activity results in decreased clearance of fatty acids within adipocytes. As a result, fatty acids can accumulate in skeletal muscle and the liver, contributing significantly to insulin resistance [110].

DM is the most common endocrinopathy of MDs, primarily due to pancreatic β-cell insufficiency secondary to mitochondrial dysfunction. Insulin deficiency is the leading cause of the hyperglycemic crisis through which DM first manifests, although insulin resistance may also occur. The first mechanism of insulin deficiency is reduced secretion resulting from impaired OXPHOS and impaired ATP supply to β-cell [111].

Chen and coworkers investigated the association between GDM and mitochondrial gene mutations. They examined mtDNA mutations from the nucleotide 3130 to 4260 encompassing tRNA^Leu^ gene and adjacent NADH dehydrogenase 1 gene in 137 patients with GDM and 292 non-diabetic pregnant controls [112]. Genetic analysis revealed that a heteroplasmic mutation T3398C was present in 2.9% of GDM patients but not in the control group, indicating an association with GDM (*P* = 0.01). Additionally, two novel mutations, a heteroplasmic C3254A and a homoplasmic A3399T, were identified in GDM subjects, warranting further investigation into their functional significance. Mutations G3316A and T3394C, previously implicated in non-insulin-dependent diabetes mellitus (NIDDM), were observed at higher frequencies in patients with GDM compared to controls [112].

In the previously mentioned De Laat’s study, a relatively high number of women (11%) had GDM in comparison to the Dutch prevalence (1,9%), with an odds ratio of 6.1 [103]. More recently, Kuleva et al. retrospectively analyzed a cohort of 75 women affected by OXPHOS disorders, both nuclear and mitochondrial, with similar results: they compared the incidence of obstetric complications with national reference data. They found a significantly higher rate of GDM (13.6 versus 3%, *P* = 0.02) and FGR (10% versus 1%, *P* = 0.008), with a fourfold increase and a tenfold increase, respectively, in a subgroup of carriers of heteroplasmic mtDNA mutations [113]. In line with previous findings, Sanchex-Lechuga and collaborators retrospectively studied the pregnancy outcomes of 26 Irish women affected by a confirmed MD (25 had a m.3243 A > G mutation in the MT-TL1 gene and 1 had the 12258 C > A mutation MT-TS2 gene). The mean heteroplasmy percentage was 18% (2–35). They found that 21/88 (24%) pregnancies were complicated by GDM, a higher rate than reported in previous studies [114], again confirming that women affected by MDs have more frequent obstetric complications, including GDM, as also documented in case reports discussed above [104, 105].

These findings suggest that mtDNA mutations might play a role in the pathogenesis of GDM. For this reason, obstetricians should take note of the possibility that a woman with MD may develop GDM.

### Polyhydramnios, oligohydramnios

The physiological volume of amniotic fluid varies, increasing as pregnancy progresses and reaching a peak of 800–1000 mL at around 36–37 weeks. Polyhydramnios, or hydramnios, is the excessive accumulation of amniotic fluid in the uterus. It occurs in 0.2–2% of pregnancies. In clinical practice, ultrasound examination is the primary method for diagnosing polyhydramnios. A finding of an amniotic fluid index (AFI) exceeding 24 centimetres (95%) or 25 centimetres (97%) is indicative of polyhydramnios. Approximately 70–80% of cases of polyhydramnios are idiopathic or caused by GDM [115], while the remaining percentage is due to fetal anomalies, aneuploidy, genetic diseases, and placental abnormalities.

Among these cases, many are attributed to open neural tube defects, central nervous system disorders impacting fetal swallowing, gastroesophageal conditions causing gastrointestinal obstruction, or conditions leading to increased urine production, such as high output hearth failure.

An AFI with an amniotic volume of less than 5 cm is called oligohydramnios [116]. Risk factors for oligohydramnios include premature rupture of membranes, intrauterine growth restriction and congenital disabilities. Oligohydramnios leads to complications in 4.4% of all pregnancies. In early pregnancies, the incidence of oligohydramnios is less than 1% [117].

In a retrospective study of 300 patients with proven OXPHOS defects, polyhydramnios was found in 6/20 (30%) of the cases and oligohydramnios in 2/20 (10%) of the cases [118]. None of the cases of amniotic fluid index anomaly was an isolated finding: polyhydramnios was associated with cardiac manifestations in 3/6 cases (rhythm anomalies, ventricular/atrial septal defects, cardiomyopathy), with gastrointestinal malformations in 2/5 cases (anal atresia, duodenal atresia) and one case was linked to hydronephrosis. In 4/6 of cases, FGR was also present. Therefore, amniotic fluid anomalies appear to be indirect manifestations of MDs and are often secondary to major fetal anomalies.

Van Rij et al. [119] reported the case of a 30-year-old primigravida, who was diagnosed with polyhydramnios at gestational week 25 + 5 (Maximal Vertical Pocket (MVP): 9,9 cm, AFI: 27.5 cm) with a normal fluid-filled abdomen and mild dilatation of both lateral ventricles (11 mm). The fetal intracranial assessment showed an enlarged cisterna magna (12 mm) and a dysplastic and small cerebellum. The myocardium was hypertrophic with a small perimembranous ventricular septal defects. A spontaneous premature birth occurred at 26 weeks; the newborn presented with facial dysmorphism (high-arched eyebrows, triangular face, up-slanted palpebral fissures, and prominent chin), hypotonia, peri- and intraventricular cerebral haemorrhage, cerebellar atrophy, lack of subcutaneous fat, ventricular septal defect, and severe lactic acidosis. The patient died two days after birth. Post-mortem genetic analysis revealed the nonsense mutations c.292 C > T (p.[Arg98*]) and c.1303 C > T (p.[Arg435*]) in *FBXL4*, a nuclear gene encoding a mitochondrial protein involved in the control of mitochondrial function. Biallelic mutations in *FBXL4* have been linked to encephalopathy associated with an mtDNA maintenance defect syndrome [119]. The authors concluded that direct testing for mutations in the *FBXL4* gene should be considered in patients with severe encephalomyopathy and polyhydramnios.

### Premature delivery

A spontaneous preterm birth (PTB) occurs when a baby is delivered naturally before the 37 week of gestation. Several studies reported higher frequencies of preterm birth in mothers affected by MD, at least for the variants m.3243 A > G [103], m.4917 A > G, m.4216T > G, m.10,398 G > A [120], and m.1555 A > G [121].

In 1997, Ewart and colleagues reported two pregnancies of a woman with Chronic Progressive External Ophthalmoplegia that resulted in preterm labours, i.e., 36th week for the first and 35th week for the second pregnancy [122]. In 2001, Okhuijsen-Kroes and coworkers described the history of a prematurely born male with lactic acidosis and hypertrophic cardiomyopathy, in whom the m.3243 A > G variant on the tRNA^Leu (UUR)^ gene was considered causative [123]. In singleton healthy pregnancies, the rate of preterm birth before 37 weeks’ gestation is around 4–5% and 1–2% before 34 weeks’ gestation.

In De Laat’s analysis, the incidence of premature delivery before 37 weeks in carriers of m.3243 A > G was reported in 23 cases (25.3%), with 5 pregnant women delivering before 32 weeks (5.5%). Compared to the prevalence in the Dutch population (7.4% for preterm birth before 37 weeks’ gestation and 1.4% before 32 weeks’ gestation), it resulted in an odd ratio of 4.2 and 4.0, respectively [103]. Kuleva et al. also reported a slight but not significant increase in the rate of PTB in women with mtDNA mutations [113].

These reports suggest that premature delivery is an occasional complication of pregnancies in women with MDs. However, further investigations are necessary to establish the correlation between MDs and the risk of preterm birth.

## Clinical manifestations of mitochondrial disease on fetal development

Antenatal manifestations reported in MDs include Fetal Growth Restriction (FGR), Subependymal pseudocysts (SEPC), Chronic intestinal pseudo-obstruction (CIPO), Cardiomyopathy (CM), reduced movement and skin oedema.

**Fig. 4:**
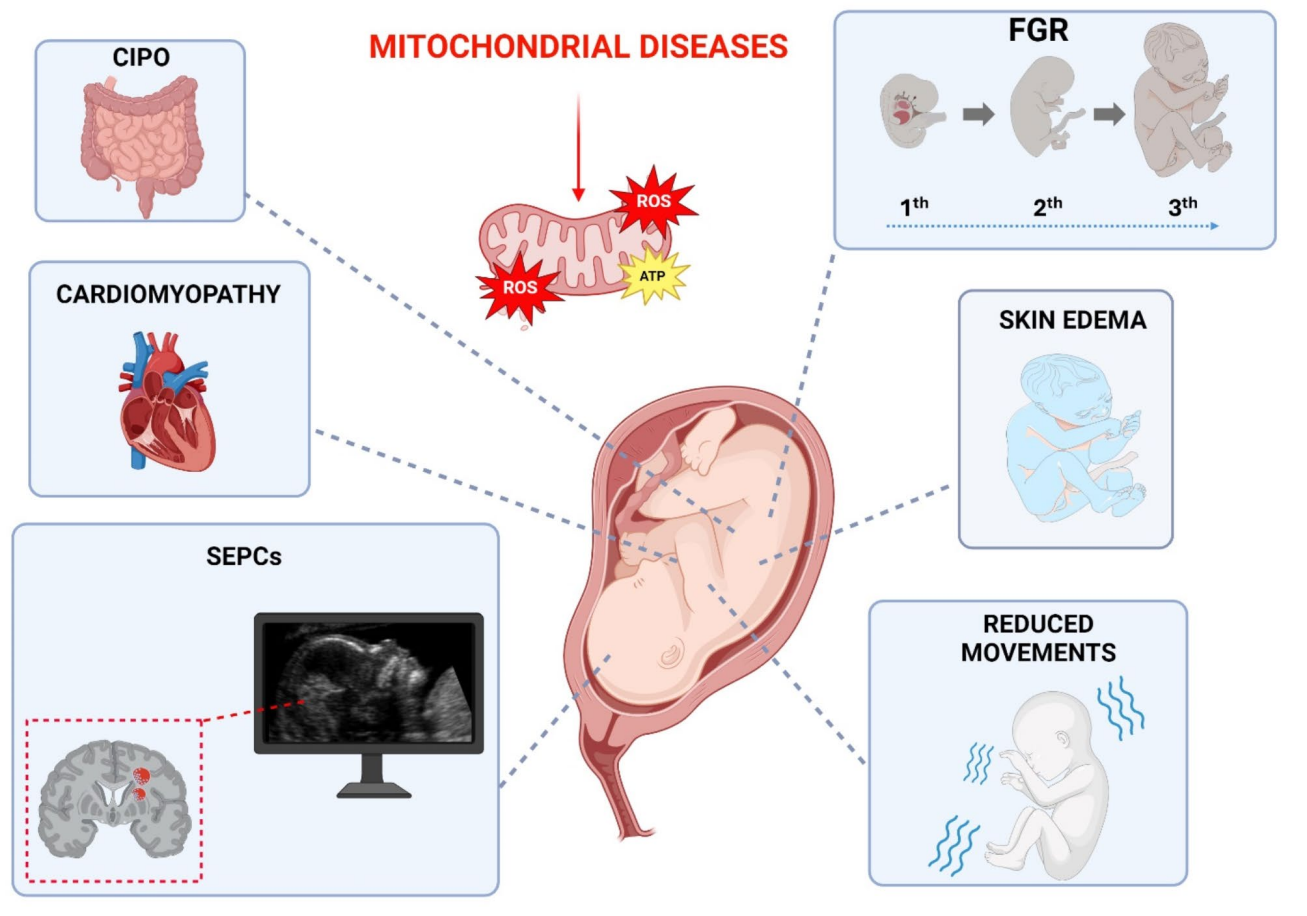
Reported antenatal presentation of MDs: Fetal Growth Restriction (FGR), Subependymal pseudocysts (SEPC), Chronic intestinal pseudo-obstruction (CIPO). See Sect. 3 for details. Created with BioRender.com

Table 3; Fig. 4 summarize the clinical manifestations of MDs in pregnancy.

**Table 3:** Clinical manifestations of MDs on fetal development

Clinical manifestation(s)	Diagnosis	Onset (GW: gestational week )	Ultrasound outcomes	Ref
*FGR and multisystemic manifestation*	Respiratory chain enzyme deficiency	After birth	FGR Reduction in fetal movements Cardiac abnormalities Gastrointestinal abnormalities CNS abnormalities	[118]
	Pyruvate dehydrogenase deficiency (PDHD); Pyruvate carboxylase deficiency (PCD)	PDHD: 25 PCD: 32	FGR Paraventricular or subependymal cysts Ventriculomegaly Macrocephaly Anomaly of gyration and corpus callosum	[127]
	Pyruvate dehydrogenase deficiency	22	FGR Paraventricular or subependymal cysts Ventriculomegaly Corpus callosum abnormalities Cerebellar hypoplasia Anomaly of gyration	[125]
*FGR and SEPCs*	Pyruvate carboxylase deficiency	33	Choroidal plexus cysts Antenatal structural anomalies and degenerative changes in the brain	[128].
	Mitochondrial depletion syndrome	19	FGR Bilateral periventricular/subependymal cysts adjacent to the frontal horns	[131]
	LS caused by mutation in the ND3 gene	38	Mild ventriculomegaly Periventricular pseudocysts	[132]
	LS caused by mutation in SURF1 gene	24	Periventricular pseudocysts	[133]
	IBA57 gene mutation	24	FGR Subependymal pseudocysts with a fluctuating membrane Dilated lateral ventricles	[134]
	NDUFAF5 biallelic variants	From 13	FGR Brain cysts Corpus callosum abnormalities Non-immune hydrops fetalis	[135]
*CIPO*	LS caused by a mutation in MT-ATP6 gene	24	Dilated hyperechogenic bowel Small volume of ascites	[139]
*Skin Oedema And Fetal Movements Reduction*	mtDNA depletion	36	Skin oedema Decrease fetal movements	[143]
*Cardiomyopathy*	OXPHOS defect involving CI and CIV	37	Biventricular hypertrophy; Pericardial effusion with a left ventricular ejection fraction of 55%	[146]
	*de novo* duplications in the *ATAD3* gene	From 26	Cardiomyopathy Perinatal death FGR Decrease fetal movements	[147]
	COX18 mutation	35	Cardiomyopathy Axonal sensory neuropathy	[148]

### FGR

Fetal growth restriction (FGR) is a multifactorial pregnancy complication associated with a high risk of in-utero or perinatal morbidity and mortality, as well as long-term adverse infant outcomes. FGR is, properly, a spectrum condition so that, beyond biometric thresholds, its prognosis relies upon gestational age and estimated fetal weight.

FGR can be isolated or associated with other structural anomalies or clinical signs of hypertensive disorders of pregnancy. The association of FGR and hypertension defines the placental phenotype called “placental insufficiency”, whose early origin in pregnancy has already been described in *Sect. 2.1.*

In a retrospective study, Von-Kleist-Retzow et al. [118] reviewed 300 cases of proven OXPHOS deficiency for fetal development based on the course and duration of pregnancy, prenatal ultrasound examination, and birth weight. Respiratory chain deficiency could have an early antenatal multiple expression affecting several organs. Notably, FGR emerged as the most frequent antenatal feature in these cases. The authors reported a prevalence of 22.7% (68/300) for low birth weight, defined as a weight at birth < 3th percentile, both isolated (48/300; 16%) and associated with multiple anomalies (20/300; 6,6%). FGR was coupled with a reduction in fetal movements, cardiac and gastrointestinal abnormalities, and central nervous system involvement such as corpus callosum agenesis, Dandy-Walker malformation, porencephalic cysts and enlarged ventricles. Although the combination of FGR and multiple organ involvement is still largely unknown, nor is it clear why only a fraction of cases manifests this feature, these observations could impact the management of at-risk pregnancies when no other prenatal clues are available [118].

A simultaneous manifestation of FGR and other prenatal anomalies in MD fetuses was also reported by Egloff et al. [124]. The authors investigated three cases, one with pyruvate dehydrogenase deficiency (PDHD) and two with pyruvate carboxylase deficiency (PCD), for which a prenatal description and ultrasound findings were available. Ultrasound anomalies, including FGR, ventriculomegaly, subependymal and periventricular cysts, did not appear until the second or third trimester. A retrospective review of the literature evaluating 11 PDHD cases and 3 PCD cases indicated that subependymal cysts associated with ventriculomegaly were indicative of PDHD, while subependymal cysts associated with macrocephaly were suggestive of PCD [124].

Among the 11 PDHD cases considered, the study of Pirot et al., 2016 is noteworthy. This report described imaging findings, clinical phenotype, and brain lesions in 4 fetuses with PDHD from 3 families with mutations in the PDHA1 or PDHB genes. Neuropathological analysis was performed in 4 autopsy cases with subsequent biochemical and molecular confirmation of PDH complex deficiency. Two cases of affected fetuses in the same family showed similar prenatal ultrasound manifestations, including FGR, paraventricular pseudocysts, ventricular dilatation, short corpus callosum and gyral abnormalities at 22–26 weeks of gestation. Both pregnancies were medically terminated [125].

In a case reported by Inbar-Feigenberg in 2018, the authors described prenatal findings of severe cerebellar growth arrest in two siblings affected by homozygous *POLG1* mutation (c.2542 G > A). The first pregnancy was complicated by symmetrical Fetal Growth Restriction polyhydramnios and abnormal repetitive fetal movements suggestive of seizures at 34th week of gestation. A premature delivery by cesarean section was planned for breech presentation at 35 + 3 weeks of gestation. The second pregnancy presented similar antenatal findings and was terminated at 19.5 weeks of gestation [126].

Mitochondria, as a primary energy source, play a crucial role in placentation and fetal development. Although FGR is quite common, affecting approximately 10% of all pregnancies, its presence, coupled with other pregnancy complications, can be an alarm signal for the possible presence of MDs in the fetus.

### SEPCs

Subependymal pseudocysts (SEPCs) are cystic cavities located at the bottom of the lateral ventricle that lack the ependymal cells typical of true cysts. They may be an isolated event that spontaneously regresses or the result of injury due to haemorrhage, intrauterine infection, or metabolic disease with impaired neuronal migration that persists after birth, as in the case of Zellweger syndrome [127]. Unusual SEPCs are diagnosed based on size, localisation and morphology.

In 2006, Garcia-Cazorla et al. retrospectively studied nine patients with the French form of pyruvate carboxylase (PC) deficiency (four females and five males) who were part of their first series of primary MDs (129 patients) diagnosed and studied in their hospital between 1977 and 2002 [128]. All patients had an early neonatal onset with severe axial hypotonia and tachypnea due to lactic acidosis as the first symptoms. In four patients, periventricular cysts were detected by brain MRI. In one of them, a prenatal cranial ultrasound showed choroidal plexus cysts at 33 weeks of pregnancy. Authors commented that cystic periventricular leukomalacia at birth was the most frequent finding in their cases [128] and, based on specific PC literature [129, 130], speculate that structural anomalies and degenerative changes in the brain would occur antenatally.

Rohrbach et al. in 2009 reported, for the first time, the prenatal ultrasound and magnetic resonance imaging findings of periventricular, large SEPCs at 19th week of gestation in a fetus diagnosed with mitochondrial DNA depletion syndrome (MDS) after birth. Delivery was induced at 37th week because of FGR, and on day 2 of life, the newborn developed tachypnea and severe lactic acidosis. Brain MRI on day 6 of life confirmed the prenatal findings with bilateral periventricular cysts and high lactate peak on magnetic resonance spectroscopy. The patient died the day after. Biochemical analysis showed decreased activities of multiple MRC complexes, suggesting a deficiency of nuclear-encoded mitochondrial enzymes. However, mutation analysis failed to detect pathogenic variants in genes *TK2*, *ANT1*, *POLG*, *SUCLA2* and *Twinkle* [131].

In 2010, Leshinsky-Silver et al. described the case of a fetus with a periventricular pseudocyst detected on brain ultrasound, in which Leigh syndrome was diagnosed postnatally. Specifically, pregnancy was uneventful until 38th week, when mild ventriculomegaly was detected by ultrasound, and a brain MRI demonstrated mildly enlarged ventricles and a periventricular pseudocyst. After birth, the newborn developed normally until the age of 3 months, when he had a sudden deterioration. A reduction in motor activity and a loss of ability to hold the head up were followed by a progressive developmental regression and ophthalmoplegia in the next months. He had elevated lactate levels in serum and cerebrospinal fluid, and urinary organic acids showed increased excretion of Krebs cycle metabolites, 3-methylglutaconic acid, lactate, and dicarboxylic acids. The features detected prenatally were confirmed with a brain MRI, and the patient died within the first year of life. Considering all the evidence, a metabolic/mitochondrial disorder was suspected, and the diagnosis was made post-mortem. Sequencing of the mtDNA revealed a heteroplasmic G10254A mutation in the ND3 gene, responsible for Leigh syndrome. The authors did not report information on FGR or other obstetric complications [132].

Cystic cerebral lesions, including SEPCs in the ventricles - based on fetal brain sonography – were also detected in 2.5% of individuals with pyruvate dehydrogenase deficiency and other mitochondrial diseases [124]. Katkevica A et al. in 2021 reported two siblings with LS due to *SURF1* pathogenic variants, i.e. c.845_846del and c.752-1G > C. For the first child, the disease appeared in the 22nd month of life with an episode of hypoglycaemia and a generally severe state of health, with an unknown intrauterine history. The mother’s second pregnancy was therefore monitored, and a cerebral periventricular cyst was detected at 24th week; fetal DNA sequencing revealed the two pathogenic variants in the *SURF1* gene in a compound heterozygous state [133].

A peculiar SEPC case in a fetus with a postnatal diagnosis of MD was reported in 2021. FGR was diagnosed at the 24th week of gestation, and few subependymal pseudocysts were observed in combination with dilated lateral ventricles at the 34th week of gestation, features that persisted postnatally. The largest cyst was located in the left ventricle, and its membrane was not tense. It showed fluctuations, which was an atypical remark. After birth, frequent apnea attacks required assisted ventilation; blood analysis detected a metabolic acidosis with a high lactate-to-pyruvate ratio. Genetic analysis revealed compound heterozygous mutations in *IBA57* gene (c.49_67dup and c.310G.T), a nuclear gene encoding a mitochondrial protein involved in the iron-sulfur cluster (4Fe-4 S) assembly. Biochemical analysis showed low mitochondrial complex I and II activities in the liver, heart, and kidney and I, II, II + III, and IV in muscle. Considering previous case reports in the literature, authors speculate that fluctuating movements in the SEPC membrane may indicate neonatal mitochondrial leukoencephalopathy [134].

Recently, Brabbing-Goldstein and colleagues described the first case series of prenatal manifestations of biallelic pathogenic variants of the *NDUFAF5* gene associated with mitochondrial CI deficiency. This multicenter retrospective study included five fetuses from three unrelated families, which shared common sonographic abnormalities such as brain cysts, corpus callosum malformations, non-immune hydrops fetalis and growth restriction [135], contrary to a previous study in which pregnancies affected by CI deficiency were reported to be mostly uneventful, possible to to the less susceptibility of fetal tissues to oxidative metabolism defects consequent to a predominant glycolytic metabolism [136]. However, the study of Brabbing-Goldestein suggested that mitochondrial CI disorders should be considered in the differential diagnosis of corpus callosum malformations and brain cysts, particularly when they are accompanied by extracranial abnormalities such as FGR and non-immune hydrops fetalis [135].

### CIPO

Chronic intestinal pseudo-obstruction (CIPO) is a rare condition characterised by symptoms of bowel obstruction in the absence of mechanical obstruction; it can be caused by a broad spectrum of disorders in children and adults, such as neurological disorders, myopathies and metabolic diseases, including MDs [137]. Since more than half of pediatric patients diagnosed with CIPO develop their symptoms in the neonatal period, very few cases of fetal CIPO were reported [138].

Among the latter, Itai T et al. reported the first case of fetal CIPO associated with LS. The case presented fetal ultrasound findings of dilated hyperechogenic bowel and a small volume of ascites at 24th week of gestation, which worsened until 31st week but then improved and finally regressed at 36th week of gestation. At six months of age, after episodes of vomiting, developmental retardation, seizure, and elevated serum level of lactate, abdominal radiography showed dilated bowel. Given that (i) CIPO is often recognised as a symptom of MDs, and (ii) brain MRI findings suggested the diagnosis of LS, mtDNA analysis was performed and revealed the presence of a mutation in MT-ATP6 gene, confirming the suspected diagnosis [139].

### Skin oedema and fetal movements reduction

Hydrops fetalis (fetal hydrops) is a pathological condition in which there is excessive accumulation of fluid in two extravascular compartments, including fetal soft tissues and body cavities; among these, skin oedema is included. Hydrops fetalis are categorized as immune and non-immune, the latter referred to as NIHF. The incidence of NIHF is estimated at 1 per 1700 to 3000 pregnancies and could be an end-stage status of many conditions [140, 141]; please refer to Bellini et al. 2015 for a complete classification [142].

In 2002, Arnon S. et al. described two sisters with diminished fetal movements and skin oedema in the third trimester but no other signs of hydrops fetalis. Within hours of birth, they developed profound lactic acidemia, followed by multi-organ failure. Both had significantly low enzymatic activity of CI, III, IV and V in muscle cells and lymphocytes. The amount of mtDNA was markedly decreased in these patients, indicative of MDS. One year after the death of the second neonate, the mother gave birth to her third daughter. The pregnancy was uneventful; there was no evidence of skin oedema, and fetal movements were regular. The physical examination was unremarkable at birth, and the biochemical investigation did not disclose any irregularity; after one year, development was appropriate for age. Since prenatal skin oedema was observed in two MDS patients but was absent in the healthy sibling, the authors suggested that skin oedema could be considered a novel manifestation of mtDNA depletion syndrome [143]. Interestingly, one year later, a retrospective study of 300 patients with confirmed MRC defects identified decreased fetal movements in 5% of them [118].

### Cardiomyopathy

Cardiomyopathy (CM) is a myocardial disorder in which the heart muscle is structurally and functionally abnormal, without other vascular dysfunction. CM is classified into (i) dilated cardiomyopathy (DCM), characterised by left ventricular dilatation and systolic dysfunction; (ii) hypertrophic cardiomyopathy (HCM), characterised by myocardial thickening; and (iii) restrictive cardiomyopathy (RCM), defined by increased myocardial stiffness, that leads to a rapid increase in ventricular pressure.

Inborn errors of metabolism account for 15–20% of all cases of pediatric CM. Among these, the most significant subgroups were MDs (8–46% of cardiomyopathy due to inborn errors of metabolism) [144]. However, only sporadic case reports and small case series have been described, so the true incidence of neonatal mitochondrial CM can only be estimated from the few reports of referral diagnostic centres. In 2005, the proportion of neonatal CM was estimated at 5,2% based on the analysis of 57 newborns with mitochondrial cytopathy [145]. In 2013, García-Diaz reported a case of a 30-year-old woman, gravida 3, whose previous baby died of congenital heart anomaly, referred at 37th week of gestation with abnormal obstetric ultrasound. Fetal echocardiographic examination showed biventricular hypertrophy and pericardial effusion with a left ventricular ejection fraction of 55%, which were confirmed at birth. Histopathological and biochemical analysis of skeletal muscle revealed a mitochondrial OXPHOS defect involving respiratory CI and IV. The authors conclude that prenatal diagnosis and fetal echocardiography can improve perinatal care due to the expected natural history and postnatal development [146]. The comprehensive assessment of the fetal and maternal condition and the standardised diagnostic procedures also provide prognostic data for genetic counselling and further potential prenatal diagnoses [146].

Recently, Frazier et al. described 17 patients with *de novo* duplications at the *ATAD3* locus, resulting in stably expressed chimeric ATAD3A/ATAD3C proteins and altered oligomerisation of the mitochondrial ATAD3 (ATPase family AAA + domain-containing member 3) protein. Affected individuals share striking clinical similarities featuring cardiomyopathy, perinatal death, and cardiac CI deficiency. For 8 of these patients (47%), prenatal analysis revealed CM in utero [147].

Recently, Ronchi and coworkers reported the first association between a mutation in COX18 and antenatal hypertrophic cardiomyopathy (detected at the 35th gestational week), followed by postnatal signs of infantile myopathy and axonal polyneuropathy with predominant affection of sensory fibres [148].

In a recent paper, Pettenuzzo and colleges [149] described newly identified compound heterozygous mutations (c.613_617delGCCGGinsCAT; p.Ala205HisfsTer48) and (c.403 A > G; p.Met135Val) in the COQ7 gene linked to hypertrophic cardiomyopathy and intestinal dysmotility, which began prenatally at 20 weeks of gestation, in two siblings from a consanguineous family. The authors described that the first affected child passed away at 14 months of age, while the clinical course in the second child was improved by supplementation with a high dose of CoQ10 (30 mg/kg/day) starting from the first days of life. In conclusion, these data indicate that early detection and genetic diagnosis are essential for defining the disease’s natural history from the pre-symptomatic stage and identifying prognostic factors. It also helps stratify patients based on their risk of organ failure, which in turn helps guide the decision to undergo a heart transplant. Finally, early detection and genetic diagnosis can lead to an appropriate monitoring and management plan being put in place at birth to prevent the disease from progressing rapidly to a fatal outcome [150].

## Influence of environmental factors on mitochondrial function and fetal development

Environmental factors, especially maternal diet and exposure to toxins, play a critical role in influencing mitochondrial function, which in turn might affect fetal development. These factors can disrupt mitochondrial DNA synthesis, repair, and overall mitochondrial function, leading to developmental abnormalities. Pregnancy increases the need for oxygen in the placenta’s mitochondria and can lead to oxidative stress, a condition where free oxygen radicals overwhelm the body’s ability to neutralize them. In certain conditions, this imbalance damages biological molecules and can cause cell death, contributing to various pregnancy complications. Factors such as socioeconomic conditions, lifestyle, diet, smoking, and environmental pollution also affect the oxidative status of pregnant women [151, 152].

While this review primarily addresses genetically determined mitochondrial diseases, it is also essential to consider the environmental factors that can induce secondary mitochondrial dysfunctions. These secondary dysfunctions may not only lead to false-positive results but also contribute to the clinical variability of MDs, thereby complicating accurate diagnosis.

### Maternal Diet and Nutrient Deficiency

The maternal diet is a primary source of nutrients essential for fetal development. Nutrients such as folate, B vitamins, and minerals like iron and magnesium are crucial for mitochondrial DNA synthesis and repair and for the proper functioning of the electron transport chain. For example, folate and vitamin B12 are vital for one-carbon metabolism, which is essential for mitochondrial DNA synthesis and methylation [153]. Deficiencies in these vitamins can impair mitochondrial function, leading to developmental defects. Omega-3 fatty acids enhance mitochondrial biogenesis and function by improving the efficiency of the electron transport chain and reducing oxidative stress, which is critical during fetal development [154]. Antioxidants like vitamins C and E help mitigate oxidative stress, a by-product of mitochondrial energy production, protecting mtDNA and proteins from damage and supporting healthy development [155].

### Environmental toxins and mitochondrial dysfunction

Exposure to environmental toxins, such as heavy metals, pesticides, and endocrine-disrupting chemicals (EDCs), can significantly impair mitochondrial function. Heavy metals like lead, mercury, and cadmium disrupt mitochondrial function by increasing oxidative stress and interfering with the electron transport chain [156]. These metals can also induce mitochondrial DNA mutations, impairing energy production and leading to developmental abnormalities. Many pesticides act as mitochondrial toxicants, inhibiting key mitochondrial enzymes, disrupting membrane potential, and increasing the production of ROS, leading to mitochondrial dysfunction and possibly negatively impacting fetal development. [157]. EDCs, including bisphenol A (BPA) and phthalates, interfere with hormone signalling pathways crucial for fetal development [158, 159]. Moreover, these chemicals can also impair mitochondrial function by disrupting calcium homeostasis and inducing oxidative stress [160].

A seminal study by Janssen et al. [161]focused on the epigenetic modifications in placental mtDNA due to environmental exposure, particularly airborne particulate matter (PM2.5). The researchers analyzed placental tissue from 381 mother-newborn pairs in the ENVIRONAGE birth cohort, measuring mtDNA methylation in the D-loop control region and 12 S rRNA (MT-RNR1), and mtDNA content by qPCR. Results showed that increased PM2.5 exposure during pregnancy is associated with higher mtDNA methylation and lower mtDNA content in placental tissue. This indicates that epigenetic changes in mtDNA, especially in the MT-RNR1 region, mediate the relationship between PM2.5 exposure and reduced mtDNA content, suggesting mechanisms of mitophagy and mitochondrial dysfunction in the early life environment.

### Mitochondrial DNA Copy Number (mtDNAcn) and air pollutants

Mitochondrial DNA copy number (mtDNAcn) indicates the size and number of mitochondria within each cell and is influenced by endogenous and environmental factors. Exposure to particulate matter (PM), particularly PM10 and PM2.5, is a strong prooxidant stimulus associated with increased mtDNAcn [162]. This increase occurs as cells exposed to oxidative stress produce more copies of their mtDNA to compensate for the damage. Alterations in mtDNAcn in various tissues, including whole blood, have emerged as potential biomarkers of mitochondrial dysfunction and risk factors for diverse chronic diseases. These disorders share oxidative stress as a common pathophysiological mechanism, which can also be highly relevant in the context of pregnancy. For example, decreased placenta mtDNAcn was observed in relation to third-trimester prenatal exposure to PM10, and altered cord blood mtDNAcn has been linked to adverse pregnancy outcomes, including abnormal fetal growth. Iodice et al. found a PM-related increase in mtDNAcn for PM10 exposure across all gestational age intervals examined. The effect was most significant during the first five weeks of pregnancy. In contrast, PM2.5 exposures were associated with increases in mtDNAcn, but these associations were not statistically significant. Moreover, the study showed a remarkable interaction between maternal BMI and mtDNAcn in relation to fetal crown-rump length (CRL) measured at the end of the first trimester, as in overweight women (BMI above 25), an inverse relationship was observed between mtDNAcn and CRL, whereas in normal-weight women, mtDNAcn was not associated with CRL [163].

Taken as a whole, these findings suggest that increased oxidative stress due to PM exposure during the first trimester of pregnancy is associated with increased mtDNAcn in maternal blood and that these changes are also linked to various fetal growth outcomes.

## Future perspectives

### In Utero fetal gene therapy

Recent advancements in gene therapy and genome editing technologies have created the potential for correcting genetic disorders in utero. Concurrently, improvements in prenatal diagnostics and fetal surgical techniques have enhanced the safety and accessibility of prenatal interventions. These technological strides have facilitated the development of protocols for in-utero treatment of various fetal conditions, such as congenital diaphragmatic hernia, myelomeningocele, pulmonary sequestration, fetal hydrothorax, and urinary tract obstructions [141]. In utero fetal gene therapy (IUFGT), therefore, is emerging as a viable therapeutic approach for addressing genetic diseases before the onset of irreversible pathology. This technique holds promise for rescuing conditions that are lethal in the perinatal period or result in significant morbidity, particularly in cases where no effective postnatal treatments exist. IUFGT is designed to deliver genetic material to specific cells in a developing fetus, leveraging various aspects of fetal development to enhance the efficiency, safety, and longevity of therapeutic delivery [164]. The small size of the fetus allows for a higher dose of gene therapy per unit of body weight, offering a significant dosing benefit and reducing the need for large quantities of viral vectors.

Additionally, certain anatomical features are more accessible during fetal development. For instance, the blood-brain barrier (BBB) is more permeable in early development, facilitating the systemic delivery of gene therapy to a wide range of central nervous system cell types and improving the efficiency of brain transduction. The presence of rapidly dividing and accessible stem/progenitor cells in multiple organs enhances the potential for the propagation and integration of therapeutic genes. Furthermore, the fetal immune system exhibits a tolerogenic profile, potentially reducing anti-vector immune responses that commonly develop postnatally, thus allowing for repeated administrations of the vector if necessary. Prenatal studies have indicated that this tolerogenic environment may be responsible for the absence of immune reactions to viral transgenes [165]. Technically, the administration of therapeutic agents into the fetus can be achieved using established techniques, such as ultrasound-guided procedures commonly utilized for umbilical vein blood transfusions in cases of fetal anemia.

The approach of IUFGT holds potential for the treatment of early-onset mitochondrial disorders, such as Leigh Syndrome (LS), a severe neurological pediatric mitochondrial disease currently lacking a cure. LS often involves mutations in the nuclear gene SURF1, which leads to defective assembly of complex IV in the mitochondrial respiratory chain, thereby impairing oxidative metabolism. Recent studies have revealed that mutations in SURF1 disrupt metabolic functions in neural progenitor cells, preventing the necessary metabolic shift from glycolysis to oxidative phosphorylation (OXPHOS). This disruption results in abnormal cell proliferation and inadequate support for neuronal morphogenesis [44]. IUFGT offers a potential therapeutic avenue for LS by enabling early intervention through the delivery of a functional SURF1 gene. Administering the wild-type gene before significant neuronal damage occurs may allow undamaged neurons to produce the necessary transgenic protein, thereby restoring OXPHOS activity and facilitating complete neural differentiation [166].

While gene therapy for MDs faces challenges due to the extensive clinical, genetic, and biochemical heterogeneity of these disorders, there is strong clinical evidence supporting the safety of in-utero gene therapy. The fetus has been shown to tolerate multiple minimally invasive procedures, such as intravascular, intraperitoneal, or intra-amniotic injections, with minimal procedural risks. These findings are promising for the future application of safe and effective prenatal treatments for lethal genetic diseases.

### Artificial Intelligence and Super Resolution Imaging

Conventional diagnostic methods for MDs, such as biochemical assays and genetic testing, have their limitations in the prenatal setting, particularly in terms of invasiveness, turnaround time, and inability to assess fetal phenotype. Antenatal ultrasound is the primary imaging technique used during pregnancy to assess the fetal health status. Accurate identification of significant fetal abnormalities, growth defects, and placental problems enables rapid and appropriate management of the pregnancy.

However, prenatal ultrasound diagnosis is associated with various challenges, such as dependence on the operator’s skills and a demanding learning process. In addition, access to skilled personnel and proper equipment is often limited in low-resource settings. Despite advances in medical imaging and fetal medicine, the diagnosis of MDs remains complex due to its rarity, genetic heterogeneity, and variable phenotypic expression. The lack of specific biomarkers and the overlap of clinical features with other disorders or environmental-acquired mitochondrial dysfunction often contribute to diagnostic delays and misdiagnosis.

The multisystem nature of MDs requires comprehensive imaging evaluation, which makes the diagnosis even more complex. The scarcity of fetal physicians with specialization in MDs further challenges routine prenatal diagnosis. Advanced ultrasonographic techniques, including sonoembryology, cardiosonography, skeletal imaging, and neurosonography, can be utilized from the earliest stages of intrauterine development.

Sonoembryology provides detailed assessments, such as the morphology of the developing heart and brain structures in embryos as small as 1–2 cm. Sonographic imaging vividly illustrates central nervous system development, cardiac function, and fetal vascularity throughout the first and second trimesters.

High-resolution ultrasonography (HRUS) employs high-frequency transducers, typically ranging from 5 to 9 MHz, along with advanced image processing techniques like harmonic imaging (HI), spatial compound imaging (SCI), and speckle reduction imaging (SRI). Compared to lower-frequency transducers (2 to 5 MHz), high-frequency transducers offer enhanced resolution, though with reduced tissue penetration, making them particularly useful for detecting subtle cardiac defects and conducting targeted neurosonographic examinations [167, 168]. HI, leveraging the nonlinear propagation of ultrasound through tissues, produces high-resolution images with minimal artefacts. In real time, SCI amalgamates multiple imaging angles into a single composite image, reducing angle-dependent artefacts. SRI diminishes speckling caused by echo interference from the ultrasound transducer [169].

Ultrasound-detected abnormalities often necessitate further evaluation using fetal magnetic resonance imaging (MRI), which aids in refining the diagnosis. Recent MRI techniques and sequences have significantly advanced our understanding of fetal brain development [170]. Three-dimensional (3D) quantitative MRI allows for exploring age-related structural dynamics and the differentiation between normal and pathological development of the fetal brain [171].

Imaging the brains of small fetuses poses considerable challenges due to the unpredictable movements of the unborn child and the difficulty in capturing small anatomical structures or distinguishing the transient layers of the developing cerebral hemispheres, especially with low tissue contrast. However, 3-Tesla (3-T) MRI scanners enhance image contrast and resolution, particularly with T2-weighted images that provide superior differentiation of anatomical features, such as water and lipid content. The increased signal-to-noise ratio from stronger gradients enables higher-resolution imaging and faster acquisition times.

Fetal MRI is frequently employed to investigate conditions such as ventriculomegaly, midline malformations, posterior fossa abnormalities, supratentorial parenchymal lesions or destruction, ischemic hemorrhagic lesions, infectious diseases, facial malformations, and neural tube defects [172]. Diffusion tensor imaging (DTI) is particularly valuable for studying axonal connectivity in the central nervous system, which is essential for understanding brain development and maturation [173]. Resting-state functional MRI (fMRI) provides insights into the functional development of the fetal brain [174]. Additionally, magnetic resonance spectroscopy (MRS) offers a non-invasive method for detecting and quantifying metabolites in the fetal brain [175, 176].

Phosphorus magnetic resonance spectroscopy (31P-MRS) and phosphorus magnetic resonance spectroscopic imaging (31P-MRSI) enable the non-invasive examination of tissue metabolism in vivo. These techniques are particularly useful for monitoring high-energy metabolites and membrane phospholipids, which are crucial for cellular energy processes. Recent applications of these methods include assessing impaired oxidative phosphorylation in the brains of adult patients with Parkinson’s disease [177] and evaluating mitochondrial oxidative energy production in skeletal muscle under exercise challenges and subsequent recovery [178]. The extension of these technologies to prenatal diagnosis holds promise for early detection of fetal signs of MDs.

Artificial Intelligence, mainly Deep Learning (DL) algorithms, has emerged as a powerful tool in medical imaging, potentially increasing diagnostic accuracy and efficiency and serving as a decision-support tool for clinicians [179]. For many common developmental disorders, AI algorithms could be trained on vast datasets of annotated ultrasound images to recognise subtle abnormalities that indicate a particular dysfunction. This would enable early identification of affected fetuses and facilitate informed counselling and management decisions. By analysing ultrasound images with unprecedented detail, AI algorithms can identify defects in neurogenesis, neuronal migration, and organogenesis that might escape human interpretation. However, the availability and quality of the training data significantly impact the technology’s effectiveness. Predictions may be inaccurate due to insufficient or biased data sets.

As with other rare diseases, the main issue currently hindering the implementation of AI for ultrasound diagnosis of MDs is the scarcity and variability of annotated datasets on which machine learning can be performed. Many animal models of MDs, such as mice [180, 181] and pigs [67] recapitulate key aspects of mitochondrial diseases and could provide a rich source of imaging data. Similarly, human organoid models derived from patient cells offer a platform for studying disease pathogenesis in vitro [182]. Animal and organoid models can serve as surrogates for human diseases and enable the generation of representative and integrative data sets. By analysing high-resolution and multimodal images (i.e. histological sections, ultrasound scan and MRI images) integrated with omics big data (i.e. genomics, spatial transcriptomics, proteomics and metabolomics), AI algorithms can uncover subtle defects (i.e. neurogenesis, neuronal migration, organogenesis), and facilitate the analysis and interpretation of complex multidimensional data. This capability may provide unprecedented insights into the early pathogenesis of mitochondrial diseases and open new avenues for prenatal diagnosis and targeted interventions.

**Fig. 5:**
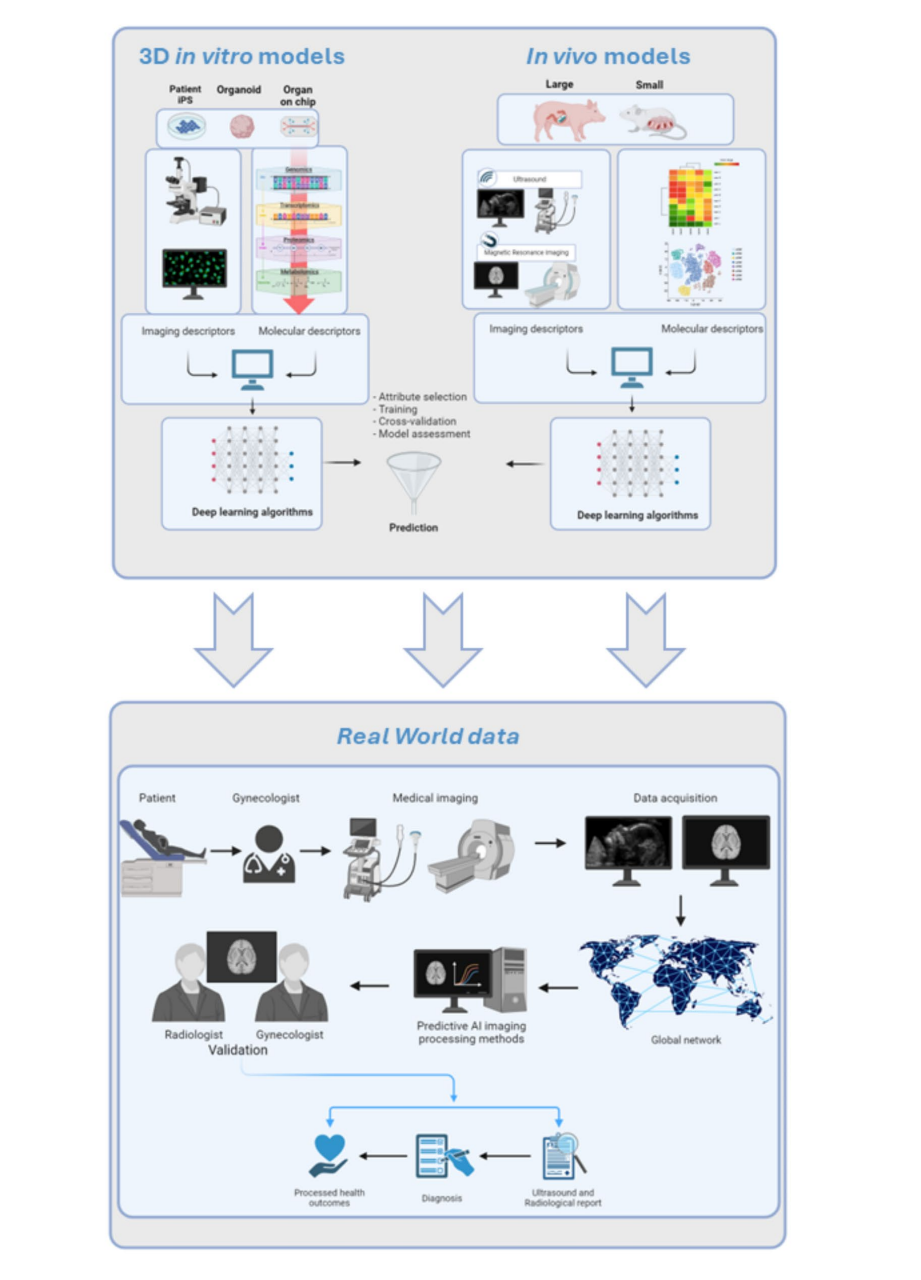
Schematic representation of translational (from preclinical to clinical) AI application for prenatal imaging identification of MDs. Created with BioRender.com

A possible approach to circumvent the above problems could be an integrated three-step approach that includes (i) a first phase of machine learning (deep learning) on preclinical models that would identify the common denominators of MDs between patient organoids and in vivo models; (ii) a second phase of machine learning that would involve searching for the outcomes identified in the DL phase in available pre-harmonised multicentre datasets; (iii) a third phase of validation of the markers in pregnancies with a history of MDs (Fig. 5).

Future research efforts should focus on refining the AI algorithms, optimising the image acquisition techniques, and validating the diagnostic criteria in different patient populations.

## Ethical issues

Prenatal diagnosis of genetic disorders involves navigating a complex array of ethical considerations. Key issues include ensuring informed consent, balancing maternal autonomy with fetal welfare, considering the implications for family planning, and ensuring equitable access to diagnostic services. It is crucial to ensure that parents fully comprehend the implications of the test results and the potential outcomes for ethical practice [183].

Mitochondrial diseases present with a broad spectrum of symptoms and severities, complicating the provision of clear and comprehensive information to prospective parents. Obstetric and genetic counselling play a critical role in helping families understand the complexities of these disorders. Effective counselling should address both the scientific aspects and the emotional and psychological impacts of potential diagnoses. The psychological effects on parents, particularly when confronted with a severe or life-limiting condition, should be carefully considered [184].

The primary objective of prenatal diagnosis of mitochondrial diseases should be to provide essential information to families about the pregnancy to optimize outcomes. In cases of severe fetal abnormalities, this information may assist families in making informed decisions regarding pregnancy termination or considering prenatal interventions.

Pre-implantation genetic diagnosis (PGD) is currently the most effective preventive strategy for families with a history of mtDNA mutations. This in vitro fertilization (IVF)-based technique involves culturing a fertilized egg containing the pathogenic mtDNA mutation until it reaches the 6–8 cell or blastocyst stage. At this point, a biopsy is performed to obtain cells for genetic analysis before implantation. However, PGD has certain limitations: (I) it is most beneficial for women with low levels of mtDNA mutations in their oocytes, and (II) it assumes that the detected heteroplasmy level accurately represents the entire embryo and remains stable over time.

Mitochondrial replacement therapy (MRT) has recently been proposed as a potential method to prevent the transmission of mutated mtDNA from mother to offspring. MRT involves replacing the mitochondria in the oocytes of women carrying mtDNA mutations with healthy mitochondria from a donor, thereby preventing the transmission of the mutation to the next generation [185]. Two primary mitochondrial replacement techniques have been proposed: maternal spindle transfer (MST) and pronuclear transfer (PNT) [186]. In MST, the spindle apparatus is extracted from an oocyte containing affected mitochondria and transferred into an enucleated donor oocyte, which is then fertilized in vitro. In PNT, an embryo is created using the intended parents’ gametes; the pronuclei are subsequently removed and transferred into an early embryo formed with the intended father’s sperm and a donor oocyte, which then continues to develop. The application of these techniques raises numerous scientific, ethical, and legal concerns, leading to diverse opinions, particularly regarding the concepts of “germline modification” and “three-parent baby” [187]. For a thorough examination of the ethical considerations associated with mitochondrial replacement therapy, refer to [188] for an extensive discussion.

In general, the ethical landscape of prenatal diagnosis for genetic diseases is uniquely complex due to debates over the moral status of the fetus and the potential use of selective abortion as a therapeutic option. These procedures aim to identify or prevent the birth of fetuses affected by undesirable traits, providing parents with choices such as conception, pregnancy termination, or managing the pregnancy as high-risk [189]. Prenatal diagnostic procedures generate extensive data and health reports, often contributing to increased uncertainty for parents. Genetic screening can exacerbate this uncertainty and raise ethical questions about the management of obtained information [190]. Concerns about the accuracy and reliability of these tests include issues such as false positives, false negatives, and the prevalence of the disorder being screened for. Key considerations include determining the necessary precision of the test in various contexts and addressing the implications of test inaccuracies.

Genetic counseling can be particularly challenging for MDs caused by mutations affecting mtDNA. Predicting the risk of disease in the child could be complicated by the inability to forecast the heteroplasmy levels in different fetal tissues. Moreover, an asymptomatic woman with low levels of mutant mtDNA may still give birth to a child severely affected by MD. As with other genetic conditions, the ethical principle of respecting maternal autonomy must be weighed against considerations of fetal welfare. In cases where MD could be diagnosed in utero, parents would be faced with the difficult decision of whether to continue or terminate the pregnancy, a decision that would require careful consideration of the potential quality of life of the child along with the personal and ethical beliefs of the mother.

The decision to terminate a pregnancy following a prenatal diagnosis of an MD would be controversial. Debates typically revolve around whether selective abortion is justified for conditions that could lead to significant suffering or if it constitutes an inappropriate interference with potential life. There is a general consensus that pregnancy termination may be justified after the second trimester if the fetus has a condition incompatible with survival or lacks cognitive function. However, the ethical considerations become more contentious when dealing with non-lethal conditions, such as many forms of MDs. Some parents may be willing to raise a child with a disability, provided they are fully informed to make an educated choice. While increased prenatal testing and subsequent terminations might reduce the incidence and prevalence of certain MDs associated with disability, this could also negatively influence public perceptions of hereditary disabilities and disabilities more broadly, potentially devaluing individuals with disabilities. The use of prenatal diagnostics primarily to prevent the birth of “disabled” babies may lead to reduced understanding and support for affected individuals and their families.

Policies on the accessibility and funding of prenatal diagnostic services for MDs may play a crucial role in ethical practice. Ensuring equitable access to these services for all individuals, irrespective of socioeconomic status, is essential. The establishment of clear guidelines for the use of prenatal diagnostic technologies and the management of sensitive genetic information is imperative. Such policies should uphold ethical standards and ensure that parents receive adequate support throughout the diagnostic process. Ongoing dialogue and research are vital to addressing these ethical challenges and supporting families facing difficult decisions regarding the prenatal diagnosis of MDs and potential interventions.

## Conclusion

The extreme clinical, genetic, and biochemical variability of MDs, coupled with the low number of patients, pose significant diagnostic challenges not only for neonatal paediatrics and neurologists but also for obstetricians due to the lack of specific knowledge about the relationship between mitochondrial diseases and pregnancy. This knowledge gap regards the foetus and the mother affected by MDs.

Several pieces of evidence collected in preclinical models revealed that severe mitochondrial dysfunction is incompatible with life or leads to critical developmental defects, highlighting the importance of correct mitochondrial function during embryo-fetal development.

However, there are only a few retrospective studies and some case reports showing that fetal anomalies or gestational disorders are clearly linked to a mitochondrial disease.

Women affected by MDs may be at increased risk of complications during pregnancy and labour, such as pre-eclampsia, gestational diabetes, polyhydramnios and preterm delivery. During fetal development, FGR, SEPCs, CIPOs, reduced fetal movement, and cardiomyopathy are the most frequent antenatal anomalies reported in cases that were diagnosed with MDs after birth.

Although several metabolic diseases undergo a symptom-free period, MDs may have an early antenatal expression, presumably related to the time course of the disease gene expression in the embryofetal period. The mechanism triggering malformations is unknown and may include an impaired metabolic switch in differentiating cells due to decreased ATP production or altered apoptotic signalling.

The lack of knowledge on the frequency of obstetric problems in MD patients and about the antenatal manifestation of MDs represents an obstacle in routine prenatal diagnosis. Establishing a surveillance procedure for MDs in utero is difficult, above all in families without a mitochondrial disease history. Consequently, most patients who receive a prenatal diagnosis of MDs have already given birth to an affected child.

MDs are diseases with significant morbidity and mortality, for which no cure exists, and effective treatments are lacking. For this reason, the identification of predictor markers could alert to the possibility of MDs and facilitate the diagnosis, not only in at-risk pregnancies but also in low-risk pregnancies. FGR, SEPCs, CIPO and skin oedema with reduced fetal movements are the principal unusual fetal ultrasound findings reported, but more data about complications during pregnancy and their frequency of manifestation are needed to establish a concrete relationship between prenatal clinical signs and MDs.

Recent advances in gene therapy and fetal medicine suggest that IUFGT could become a reality in the near future. This prenatal therapeutic perspective, however, foresees progress in prenatal diagnostic screening.

It has been stated several times that the postnatal diagnosis of MDs is not a simple process due to the extreme variability with which MDs present. Although there is currently a huge gap in knowledge on the prenatal presentation of MDs, recent advances in diagnostic imaging coupled with the development of AI algorithms could allow the identification of a certain common denominator of MDs on the fetus that represents a structurally simpler model than the pediatric or adult patient.

We believe that further research on these aspects will allowsteps forward in augmenting diagnostic accuracy and efficiency by acting as a decision-support tool for obstetricians.

## Data Availability

Not applicable.

## Abbreviations

AD: autosomal dominant
AIF: apoptosis-inducing factor
AR: autosomal recessive
ATP: adenosine triphosphate
CI: mitochondrial complex I
CIPO: chronic intestinal pseudo-obstruction
CIV: complex IV
CM: cardiomyopathy
DAMPs: damage-associated molecular patterns
DCM: dilated cardiomyopathy
DL: deep learning
DLD: dihydrolipoyl dehydrogenase
DM: diabetes mellitus
FGR: fetal growth restriction
GDM: gestational diabetes mellitus
HCM: hypertrophic cardiomyopathy
HDPs: hypertensive disorders of pregnancy
hGFAP: human glial fibrillary acidic protein
HIF-1: hypoxia-inducible factor
IPSCs: induced pluripotent stem cells
IVF: in vitro fertilisation
IVO: in vivo fertilisation
LS: leigh syndrome
MCPHA: amish lethal microcephaly
MDs: mitochondrial diseases
MRC: mitochondrial respiratory chain
MRI: magnetic resonance imaging
mtDNA: mitochondrial DNA
MtSSB: mitochondrial single-strand binding protein
NARP: neurogenic weakness, ataxia and retinitis pigmentosa
nDNA: nuclear DNA
NIHF: hydrops fetalis non-immune
NK: natural killer
NMDAS: newcastle mitochondrial disease scale for adults
NPCs: neural progenitor cells
NSCs: neural stem cells
OXPHOS: oxidative phosphorylation
PB: Preterm Birth
PC: pyruvate carboxylase
PCD: pyruvate carboxylase deficiency
PDHA1: pyruvate dehydrogenase complex E1 alpha 1 subunit
PDHc: pyruvate dehydrogenase complex
PDHD: pyruvate dehydrogenase deficiency
PE: Pre-Eclampsia
PITRM1: pitrilysin metallopeptidase 1
POLG2: polymerase γ 2
POLGA: polymerase γ A
Polγ: DNA polymerase γ
RCM: restrictive cardiomyopathy
RGCs: radial glial cells
ROS: reactive oxygen species
SEPC: subependymal pseudocysts
TFAM: mitochondrial transcription factor A
TPP: mitochondrial thiamine pyrophosphate
