# Correction to: Fetal and obstetrics manifestations of mitochondrial diseases

**DOI:** 10.1186/s12967-024-05862-9

**Published:** 2024-12-05

**Authors:** Alessia Adelizzi, Anastasia Giri, Alessia Di Donfrancesco, Simona Boito, Alessandro Prigione, Emanuela Bottani, Valentina Bollati, Valeria Tiranti, Nicola Persico, Dario Brunetti

**Affiliations:** 1 Unit of Medical Genetics and Neurogenetics, Fondazione IRCCS Istituto Neurologico “Carlo Besta”, Milan, Italy; 2 Fetal Medicine and Surgery Service, Ospedale Maggiore Policlinico, Fondazione IRCCS Ca’ Granda, Milan, Italy; 3 Department of General Pediatrics, Neonatology and Pediatric Cardiology, Medical Faculty, University Hospital Düsseldorf, Heinrich Heine University Düsseldorf, Düsseldorf, Germany; 4 Department of Diagnostics and Public Health, University of Verona, Verona, 37124 Italy; 5 Dipartimento di Scienze Cliniche e di Comunità, Dipartimento di Eccellenza, University of Milan, Milan, 2023-2027 Italy

Following publication of the original article [1], we have been notified that first and last names of authors were switched and published incorrectly.

They are now as follows:

Adelizzi Alessia^1†^, Giri Anastasia^2†^, Di Donfrancesco Alessia^1^, Boito Simona^2^, Prigione Alessandro^3^, Bottani Emanuela^4^, Bollati Valentina^5^, Tiranti Valeria^1^, Persico Nicola^2, 5^* and Brunetti Dario^1, 5^*

They should be as follows:

Alessia Adelizzi^1†^, Anastasia Giri^2†^, Alessia Di Donfrancesco^1^, Simona Boito^2^, Alessandro Prigione^3^, Emanuela Bottani^4^, Valentina Bollati^5^, Valeria Tiranti^1^, Nicola Persico^2, 5^* and Dario Brunetti^1, 5^*

The original article was updated.
